# 5-Alkylamino-*N*-phenylpyrazine-2-carboxamides: Design, Preparation, and Antimycobacterial Evaluation

**DOI:** 10.3390/molecules25071561

**Published:** 2020-03-28

**Authors:** Weronika Ambrożkiewicz, Marta Kučerová-Chlupáčová, Ondřej Janďourek, Klára Konečná, Pavla Paterová, Pavel Bárta, Jarmila Vinšová, Martin Doležal, Jan Zitko

**Affiliations:** 1 Faculty of Pharmacy in Hradec Králové, Charles University, Heyrovského 1203, 500 05 Hradec Králové, Czech Republic; kucerom@faf.cuni.cz (M.K.-C.); jando6aa@faf.cuni.cz (O.J.); konecna@faf.cuni.cz (K.K.); bartp7aa@faf.cuni.cz (P.B.); vinsova@faf.cuni.cz (J.V.); dolezalm@faf.cuni.cz (M.D.); 2 Department of Clinical Microbiology, University Hospital, Sokolská 581, 500 05 Hradec Králové, Czech Republic; pavla.paterova@fnhk.cz

**Keywords:** alkylamino derivatives, antibacterial, antifungal, antimycobacterial, cytotoxicity, pyrazinamide

## Abstract

According to the World Health Organization, tuberculosis is still in the top ten causes of death from a single infectious agent, killing more than 1.7 million people worldwide each year. The rising resistance developed by *Mycobacterium tuberculosis* against currently used antituberculars is an imperative to develop new compounds with potential antimycobacterial activity. As a part of our continuous research on structural derivatives of the first-line antitubercular pyrazinamide, we have designed, prepared, and assessed the in vitro whole cell growth inhibition activity of forty-two novel 5-alkylamino-*N*-phenylpyrazine-2-carboxamides with various length of the alkylamino chain (propylamino to octylamino) and various simple substituents on the benzene ring. Final compounds were tested against *Mycobacterium tuberculosis* H37Ra and four other mycobacterial strains (*M. aurum*, *M. smegmatis*, *M. kansasii*, *M. avium*) in a modified Microplate Alamar Blue Assay. We identified several candidate molecules with micromolar MIC against *M. tuberculosis* H37Ra and low in vitro cytotoxicity in HepG2 cell line, for example, *N*-(4-hydroxyphenyl)-5-(pentylamino)pyrazine-2-carboxamide (**3c**, MIC = 3.91 µg/mL or 13.02 µM, SI > 38) and 5-(heptylamino)-*N*-(*p*-tolyl)pyrazine-2-carboxamide (**4e**, MIC = 0.78 µg/mL or 2.39 µM, SI > 20). In a complementary screening, we evaluated the in vitro activity against bacterial and fungal strains of clinical importance. We observed no antibacterial activity and sporadic antifungal activity against the *Candida* genus.

## 1. Introduction

According to the World Health Organization (WHO) [1] recommendations, tuberculosis (TB) is already a globally established healthcare priority for which innovative new treatments are urgently needed. Human TB is mainly caused by members of the *Mycobacterium tuberculosis* complex, a group of acid-fast bacilli. It is estimated that one-quarter of the global human population (~1.7 billion of people) is sub-clinically (latently) infected by *Mycobacterium tuberculosis* (*Mtb*); however, only 5–15% of them will develop active TB disease during their lifetime [2]. In 2018, there were 10.4 million new cases of developed TB and 1.7 million deaths (including deaths from TB among HIV positives) [3]. Such statistics makes TB the number one cause of death due to the fact of a single infectious agent [4]. Due to the deficiency of rapid and sensitive diagnostic methods, an outdated vaccine (Bacille Calmette–Guérin; BCG) with limited effectiveness, and difficulty in identifying latent cases, global TB control is still questionable. Moreover, sub-treatment increases resistance to currently used antitubercular drugs [2,4]. Tuberculosis is one of the most frequent opportunistic infections for patients with human immunodeficiency virus infection and acquired immune deficiency syndrome (HIV/AIDS) and the cause of death among a quarter of them [2]. Non-tuberculous mycobacteria (NTM) can cause infection in immune-compromised individuals [4]. *Mycobacterium avium* and *Mycobacterium kansasii* belong to the subset of NTM frequently associated with opportunistic human infections, both pulmonary and extrapulmonary [5]. Disseminated NTM infections occur mainly in immunocompromised people; however, the incidence of NTM lung infections is rising globally [6]. Colonization of lungs by NTM, especially *M. avium* complex, is connected with bronchiectasis [7].

The group of first-line anti-TB drugs includes isoniazid (INH), rifampicin (RIF), ethambutol (EMB) and pyrazinamide (PZA). Pyrazinamide is an indispensable component of all basic TB treatment regimens, and it is also used in the treatment of extrapulmonary tuberculosis and pulmonary tuberculosis resistant to rifampicin or isoniazid [8].

**Figure 1:**
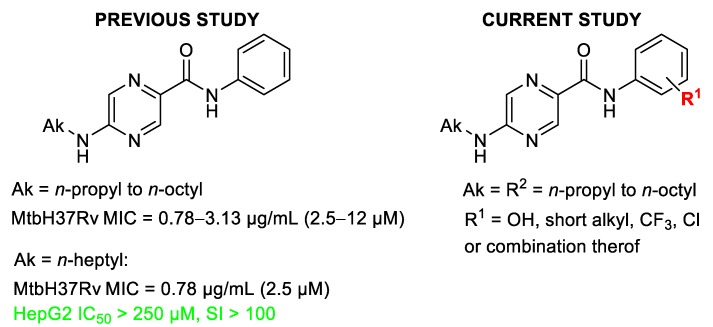
5-Alkylamino-*N*-phenylpyrazine-2-carboxamides [14] as a starting point for this study.

Many different PZA derivatives have been studied since the 1970s when PZA was established as an anti-TB drug [9]. Cynamon et al. [10] presented in vitro antimycobacterial activity of 5-chloropyrazine-2-carboxamide (5-Cl-PZA) against PZA-resistant strains and against atypical mycobacteria naturally resistant to PZA (*M. kansasii*, *M. smegmatis*, *M. fortuitum*, and *M. avium*). Our research team has already published several studies focused on structural modifications of PZA. One of our previous publication concerned a series of *N*-phenylpyrazine-2-carboxamides variously substituted on both aromatic rings which exerted significant antimycobacterial activity [11,12]. Further studies on 5-chloro-*N*-phenylpyrazine-2-carboxamides [13] and 5-alkylamino-*N*-phenylpyrazine-2-carboxamides [14] also showed promising results. Most of the tested 5-chloro-*N*-phenylpyrazine-2-carboxamides exerted activity expressed as minimum inhibitory concentration (MIC) in the range of 1.56–6.25 µg/mL [13] and alkylamino-*N*-phenylpyrazine-2-carboxamides without substitution in the phenyl ring exerted activity in the range of MIC = 0.78–3.13 µg/mL [14] (against *M. tuberculosis* H37Rv in both cases) and exerted low in vitro cytotoxicity (Figure 1, the left structure.).

The current study is an extension to the previously published series of 5-alkylamino-*N*-phenylpyrazine-2-carboxamides [14] (see the original publication for the essential points which led us to the design of this core structure). In the previous publication, we focused mainly on the role of the substituent on C-5 of the pyrazine ring along with simple alkylamino substituents, and we probed the substituents with terminal aryl, hydroxy or methoxy group in the alkylamino substituent and find out that these modifications of the alkylamino chain were mostly undesirable for antimycobacterial activity.

We now present derivatives with increased variability in the substituents on the benzene ring, including electron-donating substituents (–OH, alkyl) and electron-withdrawing substituents (–CF_3_, –Cl), both lipophilic and hydrophilic. The length of the alkylamino chain attached at position 5 in the pyrazine ring was varied the same way as in the original publication (*n*-propylamino to *n*-octylamino). In this study, we present forty-two new 5-alkylamino-*N*-phenylpyrazine-2-carboxamides, their synthesis, in vitro whole cell antimycobacterial activity, and in vitro cytotoxicity.

As a primary test, we employed the in vitro growth inhibition assay with attenuated *M. tuberculosis* (*Mtb*) H37Ra (avirulent strain). This avirulent strain is a widely used surrogate organism for the virulent *Mtb* H37Rv. It has been shown that there are only 244 non-identical proteins between *Mtb* H37Ra and *Mtb* H37Rv (virulent strain) out of more than 4000 proteins [15]. Also, the susceptibility to the majority of clinically used antimycobacterial compounds is equal or very similar between H37Ra and H37Rv [16]. These facts, together with lower costs of testing and decreased biosafety level requirements for the attenuated strain, make *Mtb* H37Ra a valuable surrogate organism for virulent *Mtb* H37Rv.

It is a common practice to use fast-growing mycobacterial species, such as *M. aurum* or *M. smegmatis*, as models for rapid screening of drugs instead of *Mtb*, which requires long incubation time and a higher biosafety level. The usage of surrogate organisms has many benefits, like being easy to handle due to the fact of its non-infectious nature and being good models of latent infection. For example, *M. aurum* can survive in macrophages and possesses drug resistance patterns and a cell wall profile similar to *M. tuberculosis* [17]. All compounds presented in this article were tested in vitro against *Mtb* H37Ra, *M. smegmatis,* and *M. aurum*. The majority of compounds were also tested against two other strains of NTM: *M. avium* and *M. kansasii*.

Additionally, final compounds were also screened for their antibacterial and antifungal activity in vitro against pathogens of clinical importance.

## 2. Results and Discussion

### 2.1. Chemistry

#### 2.1.1. Synthesis of Variously Substituted 5-chloro-*N*-phenylpyrazine-2-carboxamides as Intermediates

**Scheme 1:**
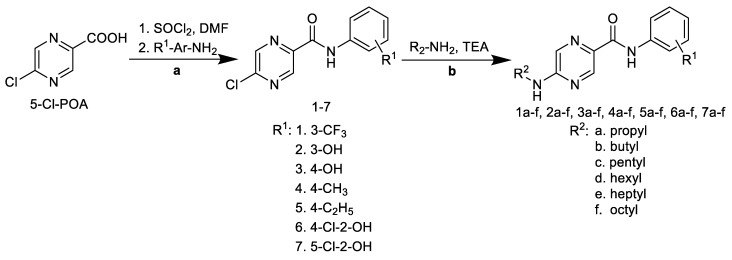
Synthesis of title compounds. Solvents and conditions: a-1—PhMe, 100 °C, 1 h; a-2—dry acetone, pyridine, RT, 1 h; b—EtOH, refluxing 7 h.

Compounds **1**–**7** (Scheme 1, a) were prepared following the previously described procedure [18] from commercially available 5-chloropyrazine-2-carboxylic acid (5-Cl-POA, Fluorochem, Hadfield, UK). The 5-Cl-POA was activated by thionyl chloride in the presence of catalytic amount of *N*,*N*-dimethylformamide to yield the acyl chloride. The crude acyl chloride was used immediately for subsequent reaction with the respective substituted aniline, using pyridine as a base. Compounds were isolated as solids in 70–90% of theoretical yield (relative to 5-Cl-POA). Five of the intermediate compounds (**1**–**3**, **5**, **6**) were reported previously [13]. Analytical data were fully consistent both with proposed structures and with previously reported values if available.

#### 2.1.2. Synthesis of Variously Substituted 5-alkylamino-*N*-phenylpyrazine-2-carboxamides

The 5-alkylmino-*N*-phenylpyrazine-2-carboxamides (**1a**–**7f**) were prepared by nucleophilic substitution of the chloropyrazine derivative by alkylamine following the previously described procedure [14] (Scheme 1, b). The relative excess (three equivalents relative to the chloropyrazine derivative) of amine was used to increase the rate of conversion. As indicated by TLC, the conversions were complete or close to complete after 3–7 h of stirring at ambient temperature. The products were purified by flash-chromatography, followed by crystallization from hot ethanol, and isolated as white, cream, beige or yellow solids with yields ranging from 15% to 98% of theoretical yield (related to the amount of starting 5-chloro-*N*-phenylpyrazine-2-carboxamide, and after all purification steps). The compounds were characterized by ^1^H-NMR, ^13^C-NMR, FT-IR spectroscopy, and melting point. The purity of the products was evaluated by elemental analysis. The analytical data were fully consistent with proposed structures. The results of the elemental analyses were in the range of ±0.5% relative to calculated values. The IR spectroscopy confirmed the presence of characteristic functional groups. In the IR spectra, the final compounds showed a signal at 1672–1639 cm^−1^, attributed to the amidic carbonyl. According to the literature data, it was expected in the regions 1750–1600 cm^−1^ (stretching) [19]. The N–H stretching bonds from the amide group and from the 5-amino group were expected in the range 3500–3000 cm^−1^ [19]. In the present case, those signals were assigned in the range 3495–3301 cm^−1^. In the ^1^H-NMR spectra (in DMSO-*d*_6_), the signal of the amidic hydrogen appeared as a singlet at 10.93–9.84 ppm. The two signals of pyrazine hydrogens H3 and H6 appeared as doublets at 7.92–8.66 ppm with coupling constant *J* in the range of 1.3–1.4 Hz which is in accordance with literature [20]. The signal of the 5-amino hydrogen appeared usually as triplet at 7.98–7.76 ppm with coupling constant *J* in the range 5.5–5.9 Hz. In the ^13^C-NMR spectra, the signal of the amidic carbon appeared at 163.46–161.64 ppm. Representative ^1^H NMR and ^13^C NMR spectra in graphic form (of selected final compounds) are attached in the Supplementary Materials.

### 2.2. Biological Activity

#### 2.2.1. Antimycobacterial Activity

All prepared compounds were screened for in vitro whole cell growth inhibitory activity against avirulent strain of Mtb H37Ra, fast growing *M. smegmatis* and *M. aurum*. Most of compounds were tested also for in vitro whole cell activity *M. kansasii* and *M. avium.* Tests were performed by modified Microplate Alamar Blue Assay (MABA) [21]. Antimycobacterial activity results were expressed as MIC in µg/mL in comparison with isoniazid (INH), rifampicin (RIF), and ciprofloxacin (CPX) as standards. The results are presented in Table 1.

Substitution of chlorine in the pyrazine ring by alkylamino substituents brought an increase in the growth inhibition of *Mtb* H37Ra. Compound **4e** showed an MIC value in the same order of magnitude as ciprofloxacin against *Mtb* H37Ra. Among alkylamino derivatives, monosubstitution on the benzene ring was more favorable (series **1**–**4**) compared to disubstituted derivatives (series **6**, **7**).

Regarding the type of substituents on the benzene ring, the highest number of active alkylamino derivatives was found in series **3** (R^1^ = 4-OH; six derivatives), followed by series **1** (R^1^ = 3-CF_3_) and **4** (R^1^ = 4-CH_3_) with three active derivatives. This finding denotes possible variability in the substituents, regarding both electronic properties (electron-donating, R^1^ = 4-OH, R^1^ = 4-CH_3_; vs. electron-withdrawing, R^1^ = 3-CF_3_) and hydrophobic properties (lipophilic versus hydrophilic). Introduction of chlorine substituent in para (series **6**) or meta (series **7**) position decreased the antimycobacterial activity. This is in concordance with previous a study, where alkylamino derivatives (C_3_-C_8_) with R^1^ = 2-Cl were inactive against *Mtb* H37Rv [14].

We also observed that minute changes in the substitution of the benzene ring led to decrease of activity in terms of higher MIC values and/or lower number of active derivatives in the series, for example, methyl versus ethyl substitution (series **4** versus **5**, homology) or 4-OH versus 3-OH (series **3** versus **2**, positional isomerism). The fact that small structural changes on the benzene ring sometimes lead to significant differences in biological activity indicates that a specific target might be involved. This is exemplified by series **3** (R^1^ = 4-OH) in which the activity grew from propylamino to octylamino substituent, in comparison with series **2** (R^1^ = 3-OH), where alkylamino derivatives longer than C_4_ were inactive. In this case, the differences in activity of positional isomers cannot be explained by modified physico-chemical properties, such as lipophilicity, which, at least by calculated log*P*, is the same. Neither in 3-OH nor 4-OH isomers do we expect any significant intramolecular H-bonds affecting the actual lipophilicity or solubility (contrary to 2-OH isomers as we showed before [13]).

In all series with monosubstitution on the benzene ring, the structural exchange of the 5-Cl of the parent compound for propylamino moiety led to preservation or increase of the activity (**1a**, **2a**, **3a**, **4a**, **5a**). In general, the propylamino substituent can be considered an activity-enhancing substituent which can introduce activity even to inactive or weakly active 5-Cl parental compounds (compare **4** versus **4a** and **5** versus **5a**).

Activity against *M. aurum* was noted only for alkylamino derivatives from series **1** (R^1^ = 3-CF_3_) with C_4_–C_7_ sidechain. The activity here culminated in pentylamino derivative **1c** with MIC = 3.13 µg/mL.

Alkylamino derivatives from series **1** (**1b**, **1c**) were active also against *M. smegmatis* with similar MIC values. Isolated activities against *M. smegmatis* were also found in series 3 (R^1^ = 4-CH_3_, **3c**, **3e**). In series **4** (R^1^ = 4-CH_3_) and **5** (R^1^ = 4-C_2_H_5_) only the propylamino derivatives (**4a**, **5a**) were active. The most successful substitution on the benzene ring enhancing the activity against *M. smegmatis* were the combinations of chloro and hydroxy as seen in series **6** (five active compounds) and **7** (four active compounds).

Compounds with activity against non-tuberculous mycobacterial strains *M. kansasii* and *M. avium* are presented in Table 2. The activity against *M. kansasii* was noted only for alkylamino derivatives with C_5_ or longer chains. The activity was equal to or higher than INH. None of the tested compounds exerted significant activity against *M. avium*.

**Figure 2:**
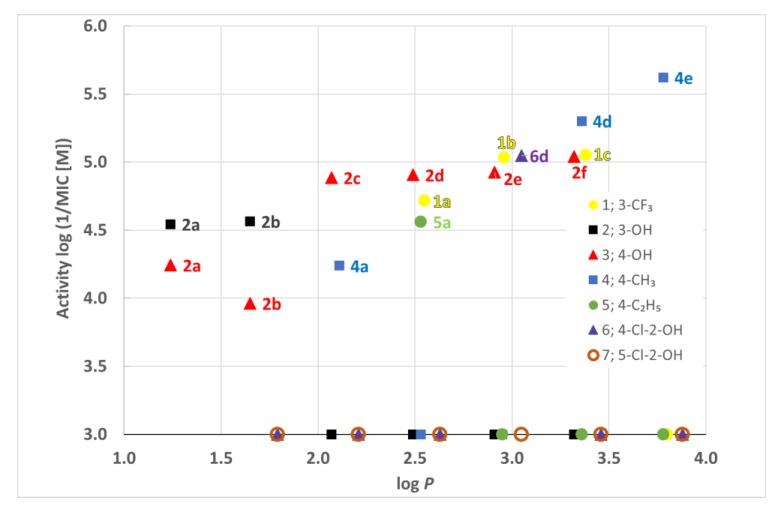
Plot of antimycobacterial activity against *Mtb* H37Ra expressed as log (1/MIC [M]) and calculated lipophilicity log*P*. Inactive compounds had an arbitrary value of activity of 3.0.

No direct correlation between calculated lipophilicity log*P* and antimycobacterial activity was observed. However, there was a slight increase in activity against *Mtb* H37Ra along with increasing lipophilicity within active compounds (Figure 2). Active compounds had log*P* values ranging from 1.24 to 3.78. Lipophilicity was not the sole determinant of antimycobacterial activity in the presented series.

#### 2.2.2. Antifungal and Antibacterial Activity

As a complementary test, final compounds were tested for in vitro activity against selected fungal and bacterial pathogens of clinical importance. The microdilution broth method was performed according to The European Committee on Antimicrobial Susceptibility Testing (EUCAST) instructions with slight modifications [22,23,24]. The minimum inhibitory concentration was expressed in µM. Not all compounds were evaluated due to the fact of a problem with solubility which occurred for 18 samples. Compounds **2c**, **2d**, **2e**, **4a**, **5b**, **5c**, and **5f** precipitated in both growth media (MHB, RPMI) and compounds **3f**, **6c**, **6d**, **6e**, **6f**, **7a**, **7b**, **7c, 7d**, **7e**, and **7f** did not dissolve in DMSO. See Supplementary Materials for the list of tested strains, methodology, and full results.

None of the evaluated compounds revealed any significant activity against the selected bacterial strains.

Five compounds revealed moderate activity against various strains of the *Candida* genus (Table 3).

#### 2.2.3. In Vitro Cytotoxicity

Results of the experiments were presented as inhibitory concentration which reduced viability of the cell population to 50% from the maximal viability (IC_50_). Cytotoxicity of the tested compounds was measured using the standard hepatic cell line HepG2. The used CellTiter 96 assay is based on the reduction of tetrazolium dye MTS in living cells to formazan, which is then determined colorimetrically. The reduction of the reagent is related to availability of NADH and NADPH. The decline in levels of these metabolically important compounds in the cell leads to reduced production of formazan.

It was not possible to determine the IC_50_ values for two-thirds of the compounds due to the fact of their low solubility in the cell culture medium. The effective cytotoxic concentrations of all tested compounds are listed in Table 1. Among these compounds, **1c** exhibited the highest cytotoxicity (IC_50_ = 18.9 µM). Other compounds with relatively high cytotoxicity were **1f**, **3d**, **3e**, **3f**, and **6c** (IC_50_ = 66.2 µM, 58.6 µM, 44.8 µM, 60.2 µM, and 81.1 µM, respectively). Compound **3c** was relatively non-cytotoxic, as its IC_50_ value was above the tested range of concentrations. Compounds **3a**, **3b**, and **5a** exhibited low cytotoxicity (IC_50_ = 410.5 µM, 377.9 µM and 494.6 µM, respectively).

## 3. Materials and Methods

### 3.1. General

Reagents and solvents were obtained from Sigma–Aldrich (Schnelldorf, Germany) if not stated otherwise and were used without further purification. The reaction progress was checked using Merck Silica 60 F_254_ TLC plates (Merck, Darmstadt, Germany). Flash chromatography of the final compounds was run on an automated chromatograph puriFlash 5.125 (INTERCHIM, Montluçon, France) using original puriFlash columns with 50 μm silica and a mixture of hexane and ethyl acetate (gradient mode), detection wavelength 254 nm. The NMR spectra were recorded on a Varian VNMR S500 (Varian, Palo Alto, CA, USA) at 500 MHz for ^1^H and 126 MHz for ^13^C. The spectra were recorded in DMSO-*d*_6_ or pyridine at ambient temperature. The chemical shifts as δ values in ppm are indirectly referenced to tetramethylsilane (TMS) via the solvent signal (2.49 for ^1^H and 39.7 for ^13^C in DMSO-*d*_6_; 8.74, 7.58, 7.22 for ^1^H and 150.35, 135.91, 123.87 for ^13^C in pyridine-*d*_5_). The IR spectra were recorded on a Nicolet Impact 400 (Nicolet, Madison, WI, USA) using the ATR-Ge method. Elemental analysis was performed on a Vario MICRO cube Element Analyzer (Elementar Analysensysteme, Hanau, Germany) and the values are given as percentages. Melting points were determined in open capillaries on a Stuart SMP20 melting point apparatus (Bibby Scientific Limited, Staffordshire, UK) and are uncorrected. Relative yields are given as percentages (of theoretical yield) and refer to the amount of product after all purification steps. The log*P* was calculated using the program CS ChemDraw Professional version 18.1.2 (PerkinElmer, Waltham, MA, USA).

### 3.2. Chemistry

#### 3.2.1. General Synthetic Procedure for Preparation of 5-chloro-*N*-phenylpyrazine-2-carboxamides

5-Chloro-*N*-phenylpyrazine-2-carboxamides (**1**–**7**) were prepared by a published procedure [13]. A mixture of 5-chloro-*N*-phenylpyrazine-2-carboxylic acid (5-Cl-POA, 10 mmol), thionyl chloride (SOCl_2_, 3 mL, 41 mmol) and *N,N*-dimethylformamide (3 drops) in dry toluene (20 mL) was stirred intensively under a reflux condenser for 1 h at 100 °C. The process turned the suspension into clear burgundy solution. After the completion of the reaction, the liquids were evaporated under reduced pressure upon addition of dry toluene (3 × 20 mL) to remove the unreacted thionyl chloride. The crude acyl chloride in the form of viscous liquid was diluted with dry acetone (20 mL) and added dropwise to the stirred and cooled (ice bath) mixture of respective aniline (9 mmol) and pyridine (1.6 mL, 20 mmol) in dry acetone (20 mL). The mixture was stirred at room temperature for 1 h and during the reaction the product precipitated from solution. Solvents were evaporated under reduced pressure and the crude product was washed with distilled water.

#### 3.2.2. General Synthetic Procedure for Preparation of 5-alkylamino-N-phenylpyrazine-2-carboxamides

The 5-alkylamino-*N*-phenylpyrazine-2-carboxamides (**1a**–**f**, **2a**–**f**, **3a**–**f**, **4a**–**f**, **5a**–**f**, **6a**–**f**, **7a**–**f**) were prepared by a published procedure [14]. A mixture of 5-chloro-*N*-phenylpyrazine-2-carboxamide (1 mmol), triethylamine (TEA, 3 mmol) and respective aliphatic amine (3 mmol) in ethanol (20 mL) was stirred intensively at 90 °C under a reflux condenser for 7 h. The progress of the reaction was monitored by TLC. To remove excess of TEA the reaction mixture was evaporated under reduced pressure with ethanol (3 × 20 mL).

Final products were purified by flash chromatography (silica, EtOAc in hexane, gradient elution) and recrystallized from hot ethanol. Prepared compounds were characterized with melting point, ^1^H NMR, ^13^C NMR, and IR spectroscopy. The purity was confirmed by elemental analysis.

### 3.3. Analytical Data of Prepared Compounds

Compounds **1**–**3**, **5**, and **6** were prepared and reported previously in a preliminary study [13] with full analytical data.

**5-Chloro-*N*-(3-(trifluoromethyl)phenyl)pyrazine-2-carboxamide (1)**

Beige solid. Yield: 86%. mp 124–125 °C (lit. mp 121.5–122.5 °C [13]). ^1^H NMR (500 MHz, DMSO-*d*_6_) δ 10.43 (s, 1H, CONH), 9.19 (d, *J* = 1.4 Hz, 1H, H3), 8.81 (d, *J* = 1.4 Hz, 1H, H6), 8.43 (s, 1H, H2′), 8.21 (d, *J* = 8.0 Hz, 1H, H4′), 7.66 (t, *J* = 8.0 Hz, 1H, H5′), 7.52 (d, *J* = 8.0 Hz, 1H, H6′). ^13^C NMR (126 MHz, DMSO-*d*_6_) δ 161.84, 152.84, 144.91, 144.04, 143.71, 139.83, 131.57 (q, *J* = 31.50 Hz), 130.70, 126.22 (q, *J* = 3.78 Hz), 124.57, 121.63 (q, *J* = 3.78 Hz), 117.59 (q, *J* = 3.78 Hz).

**5-Chloro-*N*-(3-hydroxyphenyl)pyrazine-2-carboxamide (2)**

Beige solid. Yield: 76%. mp 222–224 °C (lit. mp 225.1–226.3 °C [13]). ^1^H NMR (500 MHz, DMSO-*d*_6_) δ 10.64 (s, 1H, CONH), 9.52 (s, 1H, OH), 9.09 (d, *J* = 1.4 Hz, 1H, H3), 8.89 (d, *J* = 1.4 Hz, 1H, H6), 7.45 (t, *J* = 2.1 Hz, 1H, H2′), 7.24–7.20 (m, 1H, H4′), 7.17 (t, *J* = 8.1 Hz, 1H, H5′), 6.58–6.53 (m, 1H, H6′). ^13^C NMR (126 MHz, DMSO-*d*_6_) δ 160.90, 157.73, 151.01, 144.17, 144.14, 143.05, 139.20, 129.51, 111.69, 111.56, 107.86.

**5-Chloro-*N*-(4-hydroxyphenyl)pyrazine-2-carboxamide (3)**

Yellow solid. Yield: 70%. mp 199–201 °C (lit. mp 204.8–206.7 °C [13]). ^1^H NMR (500 MHz, DMSO-*d*_6_) δ 10.49 (s, 1H, CONH), 9.32 (s, 1H, OH), 9.06 (d, *J* = 1.4 Hz, 1H, H3), 8.87 (d, *J* = 1.4 Hz, 1H, H6), 7.67–7.58 (m, 2H, H1′, H6′), 6.77–6.69 (m, 2H, H3′, H5′). ^13^C NMR (126 MHz, DMSO-*d*_6_) δ 160.41, 154.39, 150.88, 144.32, 144.02, 143.04, 129.78, 122.51, 115.18.

**5-Chloro-*N*-(*p*-tolyl)pyrazine-2-carboxamide (4)**

Beige solid. Yield: 90%. mp 179–180 °C. ^1^H NMR (500 MHz, DMSO-*d*_6_) δ 10.63 (s, 1H, CONH), 9.09 (d, *J* = 1.4 Hz, 1H, H3), 8.91 (d, *J* = 1.4 Hz, 1H, H6), 7.76–7.73 (m, 2H, H2′, H6′), 7.18–7.15 (m, 2H, H3′, H5′), 2.28 (s, 1H, CH_3_). ^13^C NMR (126 MHz, DMSO-*d*_6_) δ 160.78, 150.98, 144.11, 144.08, 143.03, 135.70, 133.57, 129.23, 120.72, 20.69. Elemental analysis found: C, 57.85%; H, 4.11%; N, 16.88%. Calculated for C_12_H_10_N_3_O (MW 247.68): C, 58.19%; H, 4.07%; N, 16.97%.

**5-Chloro-*N*-(4-ethylphenyl)pyrazine-2-carboxamide (5)**

Beige solid. Yield: 88%. mp 156–157 °C (lit. mp 155–156.5 °C [13]). ^1^H NMR (500 MHz, DMSO-*d*_6_) δ 10.62 (s, 1H, CONH), 9.11 (d, *J* = 1.4 Hz, 1H, H3), 8.91 (d, *J* = 1.4 Hz, 1H, H6), 7.79–7.76 (m, 2H, H2′, H6′), 7.21–7.18 (m, 2H, H3′, H5′), 2.57–2.61 (m, 1H, CH_2_), 1.25 (t, *J* = 7.7 Hz, 1H, CH_3_). ^13^C NMR (126 MHz, DMSO-*d*_6_) δ 160.80, 151.00, 144.13, 144.08, 143.04, 135.89, 128.05, 120.84, 27.83, 15.86.

**5-Chloro-*N*-(5-chloro-2-hydroxyphenyl)pyrazine-2-carboxamide (6)**

Yellow solid. Yield: 73.1%. mp 220–223 °C (lit. mp 221.4–222.2 °C [13]). ^1^H NMR (500 MHz, DMSO-*d*_6_) δ 10.73 (s, 1H, CONH), 10.06 (s, 1H, OH), 9.07 (d, *J* = 1.3 Hz, 1H, H3), 8.93 (d, *J* = 1.3 Hz, 1H, H6), 8.33 (d, *J* = 2.5 Hz, 1H, H6′), 7.03 (dd, *J* = 8.7, 2.5 Hz, 1H, H4′), 6.92 (d, *J* = 8.7, 2.5 Hz, 1H, H3′). ^13^C NMR (126 MHz, DMSO-*d*_6_) δ 159.83, 151.59, 145.79, 143.69, 143.44, 142.68, 126.86, 124.27, 122.76, 118.94, 116.08. Elemental analysis found: C, 46.08%; H, 2.50%; N, 14.61%. Calculated for C_11_H_7_Cl_2_N_3_O_2_ (MW 284.10): C, 46.51%; H, 2.48%; N, 14.79%.

**5-Chloro-*N*-(4-chloro-2-hydroxyphenyl)pyrazine-2-carboxamide (7)**

Beige solid. Yield: 96%. mp 247.7–249 °C. ^1^H NMR (500 MHz, DMSO-*d*_6_) δ 10.93 (s, 1H, CONH), 10.02 (s, 1H, OH), 9.11 (s, 1H, H3), 8.94 (s, 1H, H6), 8.27 (d, *J* = 8.6 Hz, 1H, H6′), 6.96 (d, *J* = 2.3 Hz, 1H, H3′), 6.92 (dd, *J* = 8.6, 2.4 Hz, 1H, H5′). ^13^C NMR (126 MHz, DMSO-*d*_6_) δ 161.65, 156.45, 147.45, 142.92, 131.70, 131.13, 126.78, 125.98, 119.89, 119.10, 114.55. Elemental analysis found: C, 46.08%; H, 2.50%; N, 14.61%. Calculated for C_11_H_7_Cl_2_N_3_O_2_ (MW 284.10): C, 46.51%; H, 2.48%; N, 14.79%.

**5-(Propylamino)-*N*-(3-(trifluoromethyl)phenyl)pyrazine-2-carboxamide (1a)**

White solid. Yield: 87%. mp 155–156 °C. IR (ATR-Ge, cm^−1^) 3495 (N-H, NH), 3343 (N-H, CONH), 2933, 2879 (C-H, alkyl), 1670 (C=O, CONH), 1591, 1529 (C=C, Ar). ^1^H NMR (500 MHz, DMSO-*d*_6_) δ 10.48 (s, 1H, CONH), 8.66 (d, *J* = 1.3 Hz, 1H, H3), 8.40 (d, *J* = 2.1 Hz, 1H, H2′), 8.11 (dt, *J* = 7.9, 2.1 Hz, 1H, H6′), 7.97 (d, *J* = 1.3 Hz, 1H, H6), 7.91 (t, *J* = 5.6 Hz, 1H, NH), 7.55 (t, *J* = 7.9 Hz, 1H, H5′), 7.40 (dd, *J* = 7.9, 2.1, 1H, H4′), 3.33–3.27 (m, 2H, CH_2_), 1.63–1.52 (m, 2H, CH_2_), 0.92 (t, *J* = 7.4 Hz, 3H, CH_3_). ^13^C NMR (126 MHz, DMSO-*d*_6_) δ 163.17, 156.45, 143.51, 139.86, 131.49, 129.84, 129.49 (q, *J* = 31,50 Hz), 125.44, 123.78 (q, *J* = 3.78 Hz), 123.28, 119.70 (q, *J* = 3.78 Hz), 116.35 (q, *J* = 3.78 Hz), 42.27, 21.97, 11.61. Elemental analysis found: C, 55.23%; H, 4.71%; N, 17.53%. Calculated for C_15_H_15_F_3_N_4_O (MW 324.31): C, 55.55%; H, 4.66%; N, 17.28%.

**5-(Butylamino)-*N*-(3-(trifluoromethyl)phenyl)pyrazine-2-carboxamide (1b)**

White solid. Yield: 90%. mp 141–142 °C. IR (ATR-Ge, cm^−1^) 3385 (N-H, NH), 3348 (N-H, CONH), 2933, 2877 (C-H, alkyl), 1669 (C=O, CONH), 1590, 1529 (C=C, Ar). ^1^H NMR (500 MHz, DMSO-*d*_6_) δ 10.48 (s, 1H, CONH), 8.66 (d, *J* = 1.4 Hz, 1H, H3), 8.40 (d, *J* = 2.1 Hz, 1H, H2′), 8.11 (dt, *J* = 8.3, 2.1 Hz, 1H, H6′), 7.96 (d, *J* = 1.4 Hz, 1H, H6), 7.88 (t, *J* = 5.6 Hz, 1H, NH), 7.55 (t, *J* = 8.3 Hz, 1H, H5′), 7.39 (dd, *J* = 8.3, 2.1 Hz, 1H, H4′), 3.34 (td, *J* = 7.7, 5.5 Hz, 2H, CH_2_), 1.54 (dd, *J* = 7.7, 6.6 Hz, 2H, CH_2_), 1.42–1.31 (m, 2H, CH_2_), 0.90 (t, *J* = 7.3 Hz, 3H, CH_3_). ^13^C NMR (126 MHz, DMSO-d6) δ 163.17, 156.43, 143.51, 139.87, 131.46, 129.84, 129.49 (q, *J* = 31.50 Hz), 125.44, 123.77 (q, *J* = 3.78 Hz), 123.28, 119.70 (q, *J* = 3.78 Hz), 116.37 (q, *J* = 3.78 Hz), 40.15, 30.78, 19.82, 13.83. Elemental analysis found: C, 56.55%; H, 5.13%; N, 16.93%. Calculated for C_16_H_17_F_3_N_4_O (MW 338.33): C, 56.80%; H, 5.06%; N, 16.56%.

**5-(Pentylamino)-*N*-(3-(trifluoromethyl)phenyl)pyrazine-2-carboxamide (1c)**

White solid. Yield: 98%. mp 128–129 °C. IR (ATR-Ge, cm^−1^) 3307 (N-H, NH, CONH), 2933, 2875 (C-H, alkyl), 1666 (C=O, CONH), 1595, 1527 (C=C, Ar). ^1^H NMR (500 MHz, DMSO-*d*_6_) δ 10.47 (s, 1H, CONH), 8.65 (d, *J* = 1.3 Hz, 1H, H3), 8.39 (t, *J* = 2.0 Hz, 1H, H2′), 8.14–8.08 (m, 1H, H6′), 7.96 (d, *J* = 1.3 Hz ,1H, H6), 7.88 (t, *J* = 5.5 Hz, 1H, NH), 7.54 (t, *J* = 8.0 Hz, 1H, H5′), 7.42–7.36 (m, 1H, H4′), 3.33 (td, *J* = 7.1, 5.5 Hz, 2H, CH_2_), 1.55 (q, *J* = 7.1 Hz, 2H, CH_2_), 1.31 (m, 4H, (CH_2_)_2_), 0.91–0.84 (m, 3H, CH_3_). ^13^C NMR (126 MHz, DMSO-*d*_6_) δ 163.18, 156.42, 143.52, 139.88, 131.46, 129.88, 129.51 (q, *J* = 31.50 Hz), 125.45, 123.77 (q, *J* = 3.78 Hz), 123.28, 119.70 (q, *J* = 3.78 Hz), 116.35 (q, *J* = 3.78 Hz), 40.44, 28.85, 28.37, 22.06, 14.06. Elemental analysis found: C, 57.55%; H, 5.50%; N, 16.02%. Calculated for C_17_H_19_F_3_N_4_O (MW 352.36): C, 57.95%; H, 5.44%; N, 15.90%.

**5-(Hexylamino)-*N*-(3-(trifluoromethyl)phenyl)pyrazine-2-carboxamide (1d)**

White solid. Yield: 92%. mp 148–149 °C. IR (ATR-Ge, cm^−1^) 3301 (N-H, NH, CONH), 2934, 2875 (C-H, alkyl), 1665 (C=O, CONH), 1596, 1529 (C=C, Ar). ^1^H NMR (500 MHz, DMSO-*d*_6_) δ 10.48 (s, 1H, CONH), 8.65 (d, *J* = 1.4 Hz, 1H, H3), 8.40 (d, *J* = 2.1 Hz, 1H, H2′), 8.11 (dd, *J* = 8.0, 2.1 Hz, 1H, H6′), 7.96 (d, *J* = 1.4 Hz, 1H, H6), 7.88 (t, *J* = 5.6 Hz, 1H, NH), 7.54 (t, *J* = 8.0 Hz, 1H, H5′), 7.39 (dd, *J* = 8.0, 2.1 Hz, 1H, H4′), 3.36–3.29 (m, 2H, CH_2_), 1.54 (p, *J* = 7.1 Hz, 2H, CH_2_), 1.34 (m, 2H, CH_2_), 1.32–1.22 (m, 4H, (CH_2_)_2_), 0.89–0.81 (m, 3H, CH_3_). ^13^C NMR (126 MHz, DMSO-*d*_6_) δ 163.17, 156.41, 143.51, 139.87, 131.46, 129.83, 129.49 (q, *J* = 31.50 Hz), 125.45, 123.77 (q, *J* = 3.78 Hz), 123.28, 119.70 (q, *J* = 3.78 Hz), 116.34 (q, *J* = 3.78 Hz), 40.47, 31.19, 28.65, 26.34, 22.24, 14.06. Elemental analysis found: C, 58.96%; H, 5.90%; N, 15.33%. Calculated for C_18_H_21_F_3_N_4_O (MW 366.39): C, 59.01%; H, 5.78%; N, 15.29%.

**5-(Heptylamino)-*N*-(3-(trifluoromethyl)phenyl)pyrazine-2-carboxamide (1e)**

White solid. Yield: 76%. mp 148–149 °C. IR (ATR-Ge, cm^−1^) 3302 (N-H, NH, CONH), 2933, 2856 (C-H, alkyl), 1664 (C=O, CONH), 1596, 1529 (C=C, Ar). ^1^H NMR (500 MHz, DMSO-*d*_6_) δ 10.48 (s, 1H, CONH), 8.65 (d, *J* = 1.4 Hz, 1H, H3), 8.39 (t, *J* = 2.1 Hz, 1H, H2′), 8.11 (dt, *J* = 8.4, 2.1 Hz, 1H, H6′), 7.96 (d, *J* = 1.4 Hz, 1H, H6), 7.88 (t, *J* = 5.5 Hz, 1H, NH), 7.54 (t, *J* = 8.4 Hz, 1H, H5′), 7.39 (dd, *J* = 8.4, 2.1 Hz, 1H, H4′), 3.36–3.29 (m, 2H, CH_2_), 1.54 (h, *J* = 6.7 Hz, 2H, CH_2_), 1.38–1.17 (m, 8H, (CH_2_)_4_), 0.88–0.81 (m, 3H, CH_3_). ^13^C NMR (126 MHz, DMSO-*d*_6_) δ 163.46, 156.70, 143.80, 140.16, 131.74, 130.12, 129.58 (q, *J* = 31.5 Hz), 125.74, 124.06 (q, *J* = 3.78 Hz), 123.57, 120.00 (q, *J* = 3.78 Hz), 116.66 (q, *J* = 3.78 Hz), 40.75, 31.71, 28.97, 28.91, 26.92, 22.51, 14.38. Elemental analysis found: C, 59.76%; H, 6.14%; N, 14.90%. Calculated for C_19_H_23_F_3_N_4_O (MW 380.42): C, 59.99%; H, 6.09%; N, 14.73%.

**5-(Octylamino)-*N*-(3-(trifluoromethyl)phenyl)pyrazine-2-carboxamide (1f)**

White solid. Yield: 97%. mp 135–136 °C. IR (ATR-Ge, cm^−1^) 3405 (N-H, NH), 3340 (N-H, CONH), 2926, 2854 (C-H, alkyl), 1671 (C=O, CONH), 1591, 1529 (C=C, Ar). ^1^H NMR (500 MHz, DMSO-*d*_6_) δ 10.47 (s, 1H, CONH), 8.65 (d, *J* = 1.4 Hz, 1H, H3), 8.40 (t, *J* = 2.0 Hz, 1H, H2′), 8.14–8.08 (m, 1H, H6′), 7.96 (d, *J* = 1.4 Hz, 1H, H6), 7.87 (t, *J* = 5.5 Hz, 1H, NH), 7.54 (t, *J* = 8.0 Hz, 1H, H5′), 7.42–7.36 (m, 1H, H4′), 3.32 (td, *J* = 7.1, 5.5 Hz, 2H, CH_2_), 1.54 (h, *J* = 6.7 Hz, 2H, CH_2_), 1.37–1.29 (m, 2H, CH_2_), 1.28–1.20 (m, 8H, (CH_2_)_4_), 0.87–0.80 (m, 3H, CH_3_). ^13^C NMR (126 MHz, DMSO-*d*_6_) δ 163.16, 156.41, 143.50, 139.87, 131.45, 129.81, 129.49 (q, *J* = 31.50 Hz), 125.44, 123.75 (q, *J* = 3.78 Hz), 123.28, 119.68 (q, *J* = 3.78 Hz), 116.33 (q, *J* = 3.78 Hz), 40.45, 31.40, 28.91, 28.84, 28.67, 26.66, 22.24, 14.07. Elemental analysis found: C, 60.57%; H, 6.45%; N, 14.25%. Calculated for C_20_H_25_F_3_N_4_O (MW 394.44): C, 60.90%; H, 6.39%; N, 14.20%.

***N*-(3-hydroxyphenyl)-5-(propylamino)pyrazine-2-carboxamide (2a)**

White solid. Yield: 68%. mp 194–195 °C. IR (ATR-Ge, cm^−1^) 3338 (N-H, NH), 3272 (N-H, CONH), 2955, 2874 (C-H, alkyl), 1651 (C=O, CONH), 1599, 1531 (C=C, Ar). ^1^H NMR (500 MHz, DMSO-*d*_6_) δ 9.89 (s, 1H, CONH), 9.37 (s, 1H, OH), 8.63 (d, *J* = 1.4 Hz, 1H, H3), 7.95 (d, *J* = 1.4 Hz, 1H, H6), 7.84 (t, *J* = 5.6 Hz, 1H, NH), 7.43 (t, *J* = 2.2 Hz, 1H, H2′), 7.17 (dd, *J* = 8.1, 2.2 Hz, 1H, H6′), 7.08 (t, *J* = 8.0 Hz, 1H, H5′), 6.48 (dd, *J* = 8.0, 2.2 Hz, 1H, H4′), 3.30 (td, *J* = 7.3, 5.6 Hz, 2H, CH_2_), 1.57 (h, *J* = 7.3 Hz, 2H, CH_2_), 0.92 (t, *J* = 7.3 Hz, 3H, CH_3_). ^13^C NMR (126 MHz, DMSO-*d*_6_) δ 162.40, 157.72, 156.35, 143.08, 139.89, 131.93, 131.26, 129.39, 110.93, 110.71, 107.20, 42.28, 21.99, 11.64. Elemental analysis found: C, 61.83%; H, 5.97%; N, 20.24%. Calculated for C_14_H_16_N_4_O_2_ (MW 272.31): C, 61.75%; H, 5.92%; N, 20.58%.

**5-(Butylamino)pyrazine-*N*-(3-hydroxyphenyl)-2-carboxamide (2b)**

Cream solid. Yield: 84%. mp 203–204 °C. IR (ATR-Ge, cm^−1^) 3353 (N-H, NH), 3297 (N-H, CONH), 2954, 2871 (C-H, alkyl), 1655 (C=O, CONH), 1601, 1532 (C=C, Ar). ^1^H NMR (500 MHz, DMSO-*d*_6_) δ 9.89 (s, 1H, CONH), 9.37 (s, 1H, OH), 8.63 (d, *J* = 1.3 Hz, 1H, H3), 7.94 (d, *J* = 1.3 Hz, 1H, H6), 7.81 (t, *J* = 5.5 Hz, 1H, NH), 7.43 (t, *J* = 2.2 Hz, 1H, H2′), 7.17 (dd, *J* = 8.1, 2.2 Hz, 1H, H6′), 7.08 (t, *J* = 8.1 Hz, 1H, H5′), 6.48 (dt, *J* = 8.1, 2.2 Hz, 1H, H4′), 3.33 (td, *J* = 7.2, 5.5 Hz, 2H, CH_2_), 1.58–1.49 (m, 2H, CH_2_), 1.36 (h, *J* = 7.2 Hz, 2H, CH_2_), 0.90 (t, *J* = 7.2 Hz, 3H, CH_3_). ^13^C NMR (126 MHz, DMSO-*d*_6_) δ 162.41, 157.73, 156.34, 143.09, 139.90, 131.91, 131.15, 129.40, 110.93, 110.71, 107.20, 40.18, 30.82, 19.84, 13.87. Elemental analysis found: C, 63.14%; H, 6.48%; N, 19.14%. Calculated for C_15_H_18_N_4_O_2_ (MW 286.34): C, 62.92%; H, 6.34%; N, 19.57%.

***N*-(3-hydroxyphenyl)-5-(pentylamino)pyrazine-2-carboxamide (2c)**

Light cream solid. Yield: 75%. mp 224.8–226.1 °C. IR (ATR-Ge, cm^−1^) 3352 (N-H, NH), 3290 (N-H, CONH), 2934, 2873 (C-H, alkyl), 1654 (C=O, CONH), 1602, 1529 (C=C, Ar). ^1^H NMR (500 MHz, DMSO-*d*_6_) δ 9.87 (s, 1H, CONH), 9.41 (s, 1H, OH), 8.61 (d, *J* = 1.4 Hz, 1H, H3), 8.03–7.94 (m, 1H, H6), 7.94 (d, *J* = 5.6 Hz, 1H, NH), 7.41 (t, *J* = 2.2 Hz, 1H, H2′), 7.17 (dd, *J* = 8.2, 2.2 Hz, 1H, H6′), 7.07 (t, *J* = 8.2 Hz, 1H, H5′), 6.49 (dd, *J* = 8.2, 2.2 Hz, 1H, H4′), 3.32 (m, 2H, CH_2_), 2.76–2.69 (m, 2H, CH_2_), 1.55 (m, 2H, CH_2_), 1.29 (m, 2H, CH_2_), 0.86 (q, *J* = 6.7 Hz, 3H, CH_3_). ^13^C NMR (126 MHz, DMSO-*d*_6_) δ 162.40, 157.76, 156.34, 143.06, 139.86, 131.84, 131.37, 129.35, 110.88, 110.73, 107.20, 40.43, 28.86, 26.78, 22.06, 14.09. Elemental analysis found: C, 63.50%; H, 6.72%; N, 18.18%. Calculated for C_16_H_20_N_4_O_2_ (MW 300.36): C, 63.98%; H, 6.71%; N, 18.65%.

**5-(Hexylamino)pyrazine-*N*-(3-hydroxyphenyl)-2-carboxamide (2d)**

Light cream solid. Yield: 69%. mp 225–226 °C. IR (ATR-Ge, cm^−1^) 3351 (N-H, NH), 3290 (N-H, CONH), 2934, 2858 (C-H, alkyl), 1655 (C=O, CONH), 1602, 1529 (C=C, Ar). ^1^H NMR (500 MHz, DMSO-*d*_6_) δ 9.89 (s, 1H, CONH), 9.37 (s, 1H, OH), 8.62 (d, *J* = 1.4 Hz, 1H, H3), 7.94 (d, *J* = 1.4 Hz, 1H, H6), 7.82 (t, *J* = 5.5 Hz, 1H, NH), 7.42 (t, *J* = 2.2 Hz, 1H, H2′), 7.17 (dd, *J* = 8.1, 2.2 Hz, 1H, H6′), 7.08 (t, *J* = 8.1 Hz, 1H, H5′), 6.48 (dd, *J* = 8.1, 2.2 Hz, 1H, H4′), 3.32 (td, *J* = 7.1, 5.5 Hz, 2H, CH_2_), 1.54 (p, *J* = 7.1 Hz, 2H, CH_2_), 1.38–1.19 (m, 6H, (CH_2_)_3_), 0.86 (t, *J* = 6.9 Hz, 3H, CH_3_). ^13^C NMR (126 MHz, DMSO-*d*_6_) δ 162.39, 157.72, 156.32, 143.08, 139.89, 131.90, 131.19, 129.38, 110.92, 110.70, 107.19, 40.48, 31.20, 28.67, 26.35, 22.26, 14.09. Elemental analysis found: C, 65.18%; H, 7.32%; N, 17.58%. Calculated for C_17_H_22_N_4_O_2_ (MW 314.39): C, 64.95%; H, 7.05%; N, 17.82%.

**5-(Heptylamino)pyrazine-*N*-(3-hydroxyphenyl)-2-carboxamide (2e)**

Light cream solid. Yield: 47%. mp 222–223 °C. IR (ATR-Ge, cm^−1^) 3349 (N-H, NH), 3291 (N-H, CONH), 2955, 2857 (C-H, alkyl), 1653 (C=O, CONH), 1603, 1529 (C=C, Ar). ^1^H NMR (500 MHz, DMSO-*d*_6_) δ 9.89 (s, 1H, CONH), 9.36 (s, 1H, OH), 8.62 (d, *J* = 1.4 Hz, 1H, H3), 7.94 (d, *J* = 1.4 Hz, 1H, H6), 7.82 (t, *J* = 5.6 Hz, 1H, NH), 7.42 (t, *J* = 2.2 Hz, 1H, H2′), 7.17 (dd, *J* = 8.0, 2.2 Hz, 1H, H6′), 7.08 (t, *J* = 8.0 Hz, 1H, H5′), 6.48 (dd, *J* = 8.0, 2.2 Hz, 1H, H4′), 3.31 (t, *J* = 7.0 Hz, 2H, CH_2_), 1.55 (p, *J* = 7.0 Hz, 2H, CH_2_), 1.37–1.21 (m, 8H, (CH_2_)_4_), 0.85 (t, *J* = 7.0 Hz, 3H, CH_3_). ^13^C NMR (126 MHz, DMSO-*d*_6_) δ 162.39, 157.72, 156.32, 143.08, 139.89, 131.90, 131.29, 129.38, 110.91, 110.70, 107.18, 40.47, 31.43, 28.71, 28.64, 26.64, 22.23, 14.11. Elemental analysis found: C, 65.68%; H, 7.43%; N, 16.93%. Calculated for C_18_H_24_N_4_O_2_ (MW 328.42): C, 65.83%; H, 7.37%; N, 17.06%.

***N*-(3-hydroxyphenyl)-5-(octylamino)pyrazine-2-carboxamide (2f)**

Light cream solid. Yield: 65%. mp 223.0–225.9 °C. IR (ATR-Ge, cm^−1^) 3357 (N-H, NH), 3283 (N-H, CONH), 2955, 2857 (C-H, alkyl), 1652 (C=O, CONH), 1602, 1528 (C=C, Ar). ^1^H NMR (500 MHz, DMSO-*d*_6_) δ 9.89 (s, 1H, CONH), 9.37 (s, 1H, OH), 8.62 (d, *J* = 1.4 Hz, 1H, H3), 7.94 (d, *J* = 1.4 Hz, 1H, H6), 7.82 (t, *J* = 5.5 Hz, 1H, NH), 7.43 (t, *J* = 2.2 Hz, 1H, H2′), 7.17 (dd, *J* = 8.2, 2.2 Hz, 1H, H6′), 7.08 (t, *J* = 8.2 Hz, 1H, H5′), 6.48 (dd, *J* = 8.2, 2.2 Hz, 1H, H4′), 3.31 (dd, *J* = 7.1, 5.5 Hz, 2H, CH_2_), 1.54 (p, *J* = 7.1 Hz, 2H, CH_2_), 1.38–1.29 (m, 2H, CH_2_), 1.32 – 1.21 (m, 8H, (CH_2_)_4_), 0.87–0.81 (m, 3H, CH_3_). ^13^C NMR (126 MHz, DMSO-d6) δ 162.39, 157.72, 156.32, 143.08, 139.89, 131.89, 131.21, 129.38, 110.92, 110.70, 107.19, 40.47, 31.42, 28.93, 28.85, 28.71, 26.68, 22.26, 14.11. Elemental analysis found: C, 66.66%; H, 7.75%; N, 16.45%. Calculated for C_19_H_26_N_4_O_2_ (MW 342.44): C, 66.64%; H, 7.65%; N, 16.36%.

***N*-(4-hydroxyphenyl)-5-(propylamino)pyrazine-2-carboxamide (3a)**

White solid. Yield: 85%. mp 209.3–215.6 °C. IR (ATR-Ge, cm^−1^) 3307, (N-H, O-H, CONH, NH, OH), 2971 (C-H, alkyl),1638 (C=O, CONH), 1571, 1527 (C=C, Ar). ^1^H NMR (500 MHz, DMSO-*d*_6_) δ 9.85 (s, 1H, CONH), 9.21 (s, 1H, OH), 8.60 (d, *J* = 1.4 Hz, 1H, H3), 7.94 (d, *J* = 1.4 Hz, 1H, H6), 7.79 (t, *J* = 5.6 Hz, 1H, NH), 7.63–7.56 (m, 2H, H2′, H6′), 6.75–6.68 (m, 2H, H3′, H5′), 3.33–3.22 (m, 2H, CH_2_), 1.57 (h, *J* = 7.3 Hz, 2H, CH_2_), 0.92 (t, *J* = 7.3 Hz, 3H, CH_3_). ^13^C NMR (126 MHz, DMSO-*d*_6_) δ 162.04, 156.29, 153.68, 142.80, 132.19, 131.19, 130.58, 121.89, 115.17, 42.30, 22.03, 11.67. Elemental analysis found: C, 61.44%; H, 6.04%; N, 20.47%. Calculated for C_14_H_16_N_4_O_2_ (MW 272.31): C, 61.75%; H, 5.92%; N, 20.58%.

**5-(Butylamino)pyrazine-*N*-(4-hydroxyphenyl)-2-carboxamide (3b)**

White solid. Yield: 51%. mp 208.0–211.4 °C. IR (ATR-Ge, cm^−1^) 3323, (N-H, O-H, CONH, NH, OH), 2969, 2874 (C-H, alkyl),1639 (C=O, CONH), 1573, 1524 (C=C, Ar). ^1^H NMR (500 MHz, DMSO-*d*_6_) δ 9.84 (s, 1H, CONH), 9.20 (s, 1H, OH), 8.60 (d, *J* = 1.4 Hz, 1H, H3), 7.93 (d, *J* = 1.4 Hz, 1H, H6), 7.76 (t, *J* = 5.5 Hz, 1H, NH), 7.67–7.56 (m, 2H, H2′, H6′), 6.75–6.68 (m, 2H, H3′, H5′), 3.33 (td, *J* = 7.1, 5.5 Hz, 2H, CH_2_), 1.54 (p, *J* = 7.1 Hz, 2H, CH_2_), 1.36 (h, *J* = 7.1 Hz, 2H, CH_2_), 0.90 (t, *J* = 7.1 Hz, 3H, CH_3_). ^13^C NMR (126 MHz, DMSO-*d*_6_) δ 162.02, 156.26, 153.67, 142.79, 132.15, 131.26, 130.57, 121.86, 115.15, 40.16, 30.84, 19.85, 13.88. Elemental analysis found: C, 62.56%; H, 6.41%; N, 19.48%. Calculated for C_15_H_18_N_4_O_2_ (MW 286.34): C, 62.92%; H, 6.34%; N, 19.57%.

***N*-(4-hydroxyphenyl)-5-(pentylamino)pyrazine-2-carboxamide (3c)**

White solid. Yield: 76%. mp 162.6–166.8 °C. IR (ATR-Ge, cm^−1^) 3336, (N-H, O-H, CONH, NH, OH), 2956, 2869 (C-H, alkyl), 1655 (C=O, CONH), 1601, 1576, 1537, 1513 (C=C, Ar). ^1^H NMR (500 MHz, DMSO-*d*_6_) δ 9.84 (s, 1H, CONH), 9.19 (s, 1H, OH), 8.60 (d, *J* = 1.4 Hz, 1H, H3), 7.93 (d, *J* = 1.4 Hz, 1H, H6), 7.77 (t, *J* = 5.6 Hz, 1H, NH), 7.62–7.56 (m, 2H, H2′, H6′), 6.74–6.68 (m, 2H, H3′, H5′), 3.34–3.28 (m, 2H, CH_2_), 1.55 (q, *J* = 7.1 Hz, 2H, CH_2_), 1.38–1.30 (m, 4H, (CH_2_)_2_), 0.91–0.84 (m, 3H, CH_3_). ^13^C NMR (126 MHz, DMSO-*d*_6_) δ 161.99, 156.24, 153.65, 142.78, 132.15, 131.18, 130.56, 121.84, 115.13, 40.43, 28.86, 28.40, 22.07, 14.09. Elemental analysis found: C, 63.56%; H, 6.57%; N, 18.54%. Calculated for C_16_H_20_N_4_O_2_ (MW 300.36): C, 63.98%; H, 6.71%; N, 18.65%.

**5-(Hexylamino)pyrazine-*N*-(4-hydroxyphenyl)-2-carboxamide (3d)**

White solid. Yield: 34%. mp 157.7–161.5 °C. IR (ATR-Ge, cm^−1^) 3352 (N-H, NH), 3263 (N-H, O-H, CONH, OH), 2932, 2860 (C-H, alkyl),1657 (C=O, CONH), 1603, 1582, 1532, 1515 (C=C, Ar). ^1^H NMR (500 MHz, DMSO-*d*_6_) δ 9.84 (s, 1H, CONH), 9.19 (s, 1H, OH), 8.60 (d, *J* = 1.3 Hz, 1H, H3), 7.93 (d, *J* = 1.3 Hz, 1H, H6), 7.77 (t, *J* = 5.6 Hz, 1H, NH), 7.62–7.57 (m, 2H, H2′, H6′), 6.74–6.68 (m, 2H, H3′, H5′), 3.34–3.28 (m, 2H, CH_2_), 1.54 (p, *J* = 7.1 Hz, 2H, CH_2_), 1.34 (m, 2H, CH_2_), 1.27 (dt, *J* = 7.1, 3.8 Hz, 4H, (CH_2_)_2_), 0.89–0.83 (m, 3H, CH_3_). ^13^C NMR (126 MHz, DMSO-*d*_6_) δ 162.00, 156.24, 153.66, 142.78, 132.15, 131.09, 130.57, 121.84, 115.14, 40.47, 31.21, 28.69, 26.35, 22.26, 14.09. Elemental analysis found: C, 64.48%; H, 7.02%; N, 17.72%. Calculated for C_17_H_22_N_4_O_2_ (MW 314.39): C, 64.95%; H, 7.05%; N, 17.82%.

**5-(Heptylamino)pyrazine-*N*-(4-hydroxyphenyl-2-carboxamide (3e)**

White solid. Yield: 63%. mp 151.4–155.4 °C. IR (ATR-Ge, cm^−1^) 3357(N-H, NH), 3268 (N-H, O-H, CONH, OH), 2931, 2858 (C-H, alkyl), 1655 (C=O, CONH), 1602, 1582, 1531, 1513 (C=C, Ar). ^1^H NMR (500 MHz, DMSO-*d*_6_) δ 9.84 (s, 1H, CONH), 9.20 (s, 1H, OH), 8.60 (d, *J* = 1.4 Hz, 1H, H3), 7.93 (d, *J* = 1.4 Hz, 1H, H6), 7.76 (t, *J* = 5.6 Hz, 1H, NH), 7.63–7.56 (m, 2H, H2′, H6′), 6.74–6.68 (m, 2H, H3′, H5′), 3.32 (q, *J* = 6.7 Hz, 2H, CH_2_), 1.54 (p, *J* = 6.7 Hz, 2H, CH_2_), 1.37 – 1.21 (m, 8H, (CH_2_)_4_), 0.85 (t, *J* = 6.7 Hz, 3H, CH_3_). ^13^C NMR (126 MHz, DMSO-*d*_6_) δ 162.01, 156.25, 153.67, 142.79, 131.26, 132.15, 130.57, 121.85, 115.14, 40.47, 31.44, 28.74, 28.65, 26.65, 22.24, 14.12. Elemental analysis found: C, 65.51%; H, 7.40%; N, 17.07%. Calculated for C_18_H_24_N_4_O_2_ (MW 328.42): C, 65.83%; H, 7.37%; N, 17.06%.

***N*-(4-hydroxyphenyl)-5-(octylamino)pyrazine-2-carboxamide (3f)**

White solid. Yield: 60%. mp 142–143 °C. IR (ATR-Ge, cm^−1^) 3360 (N-H, NH), 3272 (N-H, O-H, CONH, OH), 2928, 2856 (C-H, alkyl), 1645 (C=O, CONH), 1601, 1582, 1529, 1512 (C=C, Ar). ^1^H NMR (500 MHz, DMSO-*d*_6_) δ 9.84 (s, 1H, CONH), 9.19 (s, 1H, OH), 8.60 (d, *J* = 1.3 Hz, 1H, H3), 7.93 (d, *J* = 1.3 Hz, 1H, H6), 7.76 (t, *J* = 5.5 Hz, 1H, NH), 7.63 – 7.56 (m, 2H, H2′, H6′), 6.75 – 6.67 (m, 2H, H3′, H5′), 3.31 (q, *J* = 6.8 Hz, 2H, CH_2_), 1.54 (p, *J* = 6.8 Hz, 2H, CH_2_), 1.33 (h, *J* = 6.8 Hz, 2H, CH_2_), 1.31 – 1.18 (m, 8H, (CH_2_)_4_), 0.87 – 0.81 (m, 3H, CH_3_). ^13^C NMR (126 MHz, DMSO-*d*_6_) δ 161.99, 156.24, 153.66, 142.77, 132.14, 131.16, 130.57, 121.83, 115.13, 40.46, 31.42, 28.93, 28.85, 28.71, 26.68, 22.26, 14.11. Elemental analysis found: C, 66.77%; H, 7.73%; N, 16.26%. Calculated for C_19_H_26_N_4_O_2_ (MW 342.44): C, 66.64%; H, 7.65%; N, 16.36%.

**5-(Propylamino)-*N*-(*p*-tolyl)pyrazine-2-carboxamide (4a)**

Cream solid. Yield: 83%. mp 168–169 °C. IR (ATR-Ge, cm^−1^) 3356 (N-H, NH), 3290 (N-H, CONH), 2927, 2875 (C-H, alkyl), 1655 (C=O, CONH), 1591, 1518 (C=C, Ar). ^1^H NMR (500 MHz, DMSO-*d*_6_) δ 9.98 (s, 1H, CONH), 8.63 (d, *J* = 1.3 Hz, 1H, H3), 7.95 (d, *J* = 1.3 Hz, 1H, H6), 7.83 (t, *J* = 5.6 Hz, 1H, NH), 7.74 – 7.68 (m, 2H, H2′, H6′), 7.12 (d, *J* = 8.2 Hz, 2H, H3′, H5′), 3.34–3.26 (m, 2H, CH_2_), 2.26 (s, 3H, CH_3_), 1.57 (h, *J* = 7.3 Hz, 2H, CH_2_), 0.92 (t, *J* = 7.3 Hz, 3H, CH_3_). ^13^C NMR (126 MHz, DMSO-*d*_6_) δ 162.38, 156.34, 143.02, 136.42, 132.45, 131.98, 131.04, 129.15, 120.10, 42.28, 22.00, 20.64, 11.65. Elemental analysis found: C, 66.81%; H, 6.72%; N, 20.61%. Calculated for C_15_H_18_N_4_O (MW 270.34): C, 66.64%; H, 6.71%; N, 20.73%.

**5-(Butylamino)-*N*-(*p*-tolyl)pyrazine-2-carboxamide (4b)**

Yellow crystalline. Yield: 32%. mp 162.6–163.6 °C. IR (ATR-Ge, cm^−1^) 3358 (N-H, NH), 3268 (N-H, CONH), 2956, 2869 (C-H, alkyl), 1667 (C=O, CONH), 1591, 1570, 1517 (C=C, Ar). ^1^H NMR (500 MHz, Pyridine-*d*_5_) δ 10.27 (s, 1H, CONH), 9.28 (d, *J* = 1.4 Hz, 1H, H3), 8.36 (t, *J* = 5.6 Hz, 1H, NH), 8.13–8.07 (m, 2H, H2′, H6′), 7.96 (d, *J* = 1.4 Hz, 1H, H6), 7.22–7.17 (m, 2H, H3′, H5′), 3.51 (q, *J* = 7.4 Hz, 2H, CH_2_), 2.22 (s, 3H, CH_3_), 1.66–1.56 (m, 2H, CH_2_), 1.34 (h, *J* = 7.4 Hz, 2H, CH_2_), 0.84 (t, *J* = 7.4 Hz, 3H, CH_3_). ^13^C NMR (126 MHz, Pyridine-*d*_5_) δ 163.02, 157.12, 143.87, 137.21, 133.17, 132.93, 130.87, 129.80, 120.38, 41.10, 31.48, 20.78, 20.43, 13.89. Elemental analysis found: C, 67.57%; H, 7.18%; N, 19.63%. Calculated for C_16_H_20_N_4_O (MW 284.36): C, 67.58%; H, 7.09%; N, 19.70%.

**5-(Pentylamino)-*N*-(*p*-tolyl)pyrazine-2-carboxamide (4c)**

Cream solid. Yield: 52%. mp 155.9–156.6 °C. IR (ATR-Ge, cm^−1^) 3344 (N-H, NH), 3283 (N-H, CONH), 2929, 2872 (C-H, alkyl), 1654 (C=O, CONH), 1590, 1547, 1519 (C=C, Ar). ^1^H NMR (500 MHz, DMSO-*d*_6_) δ 9.98 (s, 1H, CONH), 8.64–8.60 (m, 1H, H3), 7.95 (d, *J* = 1.4 Hz, 1H, H6), 7.82 (t, *J* = 5.5 Hz, 1H, NH), 7.74–7.68 (m, 2H, H2′, H6′), 7.12 (d, *J* = 8.3 Hz, 2H, H3′, H5′), 3.36–3.29 (m, 2H, CH_2_), 2.26 (s, 3H, CH_3_), 1.60–1.51 (m, 2H, CH_2_), 1.37–1.26 (m, *J* = 3.8, 3.2 Hz, 4H, (CH_2_)_2_), 0.91–0.84 (m, 3H, CH_3_). ^13^C NMR (126 MHz, DMSO-*d*_6_) δ 162.37, 156.30, 143.02, 136.42, 132.43, 131.95, 131.05, 129.14, 120.09, 40.44, 28.86, 28.39, 22.06, 20.63, 14.08. Elemental analysis found: C, 68.95%; H, 7.49%; N, 18.94%. Calculated for C_17_H_22_N_4_O (MW 298.39): C, 68.43%; H, 7.43%; N, 18.78%.

**5-(Hexylamino)-*N*-(*p*-tolyl)pyrazine-2-carboxamide (4d)**

Cream solid. Yield: 66%. mp 163–164 °C. IR (ATR-Ge, cm^−1^) 3344 (N-H, NH), 3287 (N-H, CONH), 2926, 2857 (C-H, alkyl), 1655 (C=O, CONH), 1590, 1547, 1520 (C=C, Ar). ^1^H NMR (500 MHz, DMSO-*d*_6_) δ 9.98 (s, 1H, CONH), 8.62 (d, *J* = 1.4 Hz, 1H, H3), 7.94 (d, *J* = 1.4 Hz, 1H, H6), 7.81 (t, *J* = 5.5 Hz, 1H, NH), 7.74–7.68 (m, 2H, H2′, H6′), 7.12 (d, *J* = 8.3 Hz, 2H, H3′, H5′), 3.36–3.28 (m, 2H, CH_2_), 2.26 (s, 3H, CH_3_), 1.59–1.50 (m, 2H, CH_2_), 1.38–1.22 (m, 6H, (CH_2_)_3_), 0.91–0.83 (m, 3H, CH_3_). ^13^C NMR (126 MHz, DMSO-*d*_6_) δ 162.36, 156.30, 143.01, 136.42, 132.43, 131.95, 131.27, 129.14, 120.08, 40.48, 31.20, 28.68, 26.35, 22.25, 20.63, 14.08. Elemental analysis found: C, 69.32%; H, 7.84%; N, 17.83%. Calculated for C_18_H_24_N_4_O (MW 312.42): C, 69.20%; H, 7.74%; N, 17.93%.

**5-(Heptylamino)-*N*-(*p*-tolyl)pyrazine-2-carboxamide (4e)**

White solid. Yield: 38%. mp 157.8–158.9 °C. IR (ATR-Ge, cm^−1^) 3354 (N-H, NH), 3292 (N-H, CONH), 2928, 2856 (C-H, alkyl), 1659 (C=O, CONH), 1592, 1521 (C=C, Ar). ^1^H NMR (500 MHz, DMSO-*d*_6_) δ 9.98 (s, 1H, CONH), 8.61 (d, *J* = 1.4 Hz, 1H, H3), 7.98 (d, *J* = 1.4 Hz, 1H, H6), 7.94 (t, *J* = 5.5 Hz, 1H, NH), 7.74 – 7.69 (m, 2H, H2′, H6′), 7.11 (d, *J* = 8.2 Hz, 2H, H3′, H5′), 3.35–3.29 (m, 2H, CH_2_), 2.26 (s, 3H, CH_3_), 1.59–1.49 (m, 2H, CH_2_), 1.37–1.18 (m, 8H, (CH_2_)_4_), 0.85 (td, *J* = 6.9, 2.1 Hz, 3H, CH_3_). ^13^C NMR (126 MHz, DMSO-*d*_6_) δ 162.38, 156.33, 142.99, 136.43, 132.41, 131.88, 131.22, 129.13, 120.08, 40.44, 31.33, 28.68, 27.12, 26.82, 22.18, 20.63, 14.09. Elemental analysis found: C, 69.61%; H, 8.08%; N, 17.07%. Calculated for C_19_H_26_N_4_O (MW 326.44): C, 69.91%; H, 8.03%; N, 17.16%.

**5-(octylamino)-*N*-(*p*-tolyl)pyrazine-2-carboxamide (4f)**

Cream solid. Yield: 66%. mp 155–156 °C. IR (ATR-Ge, cm^−1^) 3343 (N-H, NH), 3279 (N-H, CONH), 2928, 2854 (C-H, alkyl), 1655 (C=O, CONH). ^1^H NMR (500 MHz, Pyridine-*d*_5_) δ 10.27 (s, 1H, CONH), 9.30 (d, *J* = 1.2 Hz, 1H, H3), 8.40 (t, *J* = 5.6 Hz, 1H, NH), 8.13–8.07 (m, 2H, H2′, H6′), 7.99 (d, *J* = 1.2 Hz, 1H, H6), 7.19 (d, *J* = 8.2 Hz, 2H, H3′, H5′), 3.55 (q, *J* = 7.36 Hz, 2H, CH_2_), 2.22 (s, 3H, CH_3_), 1.67 (p, *J* = 7.3 Hz, 2H, CH_2_), 1.39–1.29 (m, 2H, CH_2_), 1.27–1.11 (m, 8H, (CH_2_)_4_), 0.83 (td, *J* = 7.3, 1.0 Hz, 3H, CH_3_). ^13^C NMR (126 MHz, Pyridine-*d*_5_) δ 163.02, 157.16, 143.89, 137.21, 133.17, 132.95, 130.91, 129.80, 120.37, 41.46, 31.97, 29.54, 29.49, 29.46, 27.36, 22.85, 20.79, 14.23. Elemental analysis found: C, 70.27%; H, 8.42%; N, 16.37%. Calculated for C_20_H_28_N_4_O (MW 340.47): C, 70.56%; H, 8.29%; N, 16.46%.

***N*-(4-ethylphenyl)-5-(propylamino)pyrazine-2-carboxamide (5a)**

Cream solid. Yield: 69%. mp 141.8–142.8 °C. IR (ATR-Ge, cm^−1^) 3355 (N-H, NH), 3283 (N-H, CONH), 2964, 2827 (C-H, alkyl), 1651 (C=O, CONH), 1589, 1548, 1518 (C=C, Ar). ^1^H NMR (500 MHz, DMSO-*d*_6_) δ 9.99 (s, 1H, CONH), 8.63 (d, *J* = 1.3 Hz, 1H, H3), 7.95 (d, *J* = 1.3 Hz, 1H, H6), 7.84 (t, *J* = 5.6 Hz, 1H, NH), 7.77–7.70 (m, 2H, H2′, H6′), 7.18–7.12 (m, 2H, H3′, H5′), 3.30 (td, *J* = 7.3, 5.6 Hz, 2H, CH_2_), 2.56 (q, *J* = 7.6 Hz, 2H, CH_2_), 1.57 (h, *J* = 7.3 Hz, 2H, CH_2_), 1.16 (t, *J* = 7.6 Hz, 3H, CH_3_), 0.93 (t, *J* = 7.3 Hz, 3H, CH_3_). ^13^C NMR (126 MHz, DMSO-*d*_6_) δ 162.36, 156.33, 143.01, 138.92, 136.60, 131.97, 131.21, 127.94, 120.18, 42.27, 27.79, 21.99, 15.87, 11.63. Elemental analysis found: C, 67.55%; H, 7.17%; N, 19.59%. Calculated for C_16_H_20_N_4_O (MW 284.36): C, 67.58%; H, 7.09%; N, 19.70%.

**5-(Butylamino)pyrazine-*N*-(4-ethylphenyl)-2-carboxamide (5b)**

Cream solid. Yield: 74%. mp 133.8–134.8 °C. IR (ATR-Ge, cm^−1^) 3354 (N-H, NH), 3265 (N-H, CONH), 2955, 2871 (C-H, alkyl), 1664 (C=O, CONH), 1590, 1573, 1519 (C=C, Ar). ^1^H NMR (500 MHz, DMSO-*d*_6_) δ 9.99 (s, 1H, CONH), 8.63 (d, *J* = 1.3 Hz, 1H, H3), 7.95 (d, *J* = 1.3 Hz, 1H, H6), 7.81 (t, *J* = 5.5 Hz, 1H, NH), 7.77–7.70 (m, 2H, H2′, H6′), 7.17–7.12 (m, 2H, H3′, H5′), 3.37–3.30 (m, 2H, CH_2_), 2.56 (q, *J* = 7.6 Hz, 2H, CH_2_), 1.59–1.49 (m, 2H, CH_2_), 1.36 (h, *J* = 7.3 Hz, 2H, CH_2_), 1.16 (t, *J* = 7.6 Hz, 3H, CH_3_), 0.90 (t, *J* = 7.3 Hz, 3H, CH_3_). ^13^C NMR (126 MHz, DMSO-*d*_6_) δ 162.36, 156.31, 143.02, 138.91, 136.60, 131.94, 131.20, 127.94, 120.17, 40.15, 30.81, 27.79, 19.83, 15.87, 13.85. Elemental analysis found: C, 68.38%; H, 7.63%; N, 18.69%. Calculated for C_17_H_22_N_4_O (MW 298.39): C, 68.43%; H, 7.43%; N, 18.78%.

***N*-(4-ethylphenyl)-5-(pentylamino)pyrazine-2-carboxamide (5c)**

Cream/white solid. Yield: 58%. mp 136.1–137.1 °C. IR (ATR-Ge, cm^−1^) 3346 (N-H, NH), 3296 (N-H, CONH), 2930, 2871 (C-H, alkyl), 1662 (C=O, CONH), 1592, 1520 (C=C, Ar). ^1^H NMR (500 MHz, DMSO-*d*_6_) δ 9.99 (s, 1H, CONH), 8.63 (d, *J* = 1.3 Hz, 1H, H3), 7.95 (d, *J* = 1.3 Hz, 1H, H6), 7.81 (t, *J* = 5.5 Hz, 1H, NH), 7.78–7.70 (m, 2H, H2′, H6′), 7.18–7.12 (m, 2H, H3′, H5′), 3.36–3.29 (m, 2H, CH_2_), 2.56 (q, *J* = 7.6 Hz, 2H, CH_2_), 1.60–1.51 (m, 2H, CH_2_), 1.38–1.27 (m, 4H, (CH_2_)_2_), 1.16 (dd, *J* = 8.0, 7.2 Hz, 3H, CH_3_), 0.91–0.84 (m, 3H, CH_3_). ^13^C NMR (126 MHz, DMSO-*d*_6_) δ 162.37, 156.30, 143.02, 138.92, 136.61, 131.95, 131.25, 127.95, 120.18, 40.44, 28.86, 28.39, 27.80, 22.07, 15.88, 14.08. Elemental analysis found: C, 69.25%; H, 7.83%; N, 17.83%. Calculated for C_18_H_24_N_4_O (MW 312.42): C, 69.20%; H, 7.74%; N, 17.93%.

***N*-(4-ethylphenyl)-5-(hexylamino)pyrazine-2-carboxamide (5d)**

Cream solid. Yield: 82%. mp 138–139 °C. IR (ATR-Ge, cm^−1^) 3336 (N-H, NH), 3293 (N-H, CONH), 2929, 2869 (C-H, alkyl), 1662 (C=O, CONH), 1592, 1548, 1519 (C=C, Ar). ^1^H NMR (500 MHz, DMSO-*d*_6_) δ 9.99 (s, 1H, CONH), 8.63 (d, *J* = 1.3 Hz, 1H, H3), 7.95 (d, *J* = 1.3 Hz, 1H, H6), 7.81 (t, *J* = 5.5 Hz, 1H, NH), 7.76–7.70 (m, 2H, H2′, H6′), 7.18–7.11 (m, 2H, H3′, H4′), 3.36–3.29 (m, 2H, CH_2_), 2.56 (q, *J* = 7.6 Hz, 2H, CH_2_), 1.59–1.50 (m, 2H, CH_2_), 1.37–1.22 (m, 6H, (CH_2_)_3_), 1.16 (t, *J* = 7.6 Hz, 3H, CH_3_), 0.90–0.82 (m, 3H, CH_3_). ^13^C NMR (126 MHz, DMSO-*d*_6_) δ 162.36, 156.29, 143.01, 138.91, 136.60, 131.93, 131.23, 127.94, 120.17, 40.47, 31.19, 28.67, 27.79, 26.34, 22.25, 15.86, 14.07. Elemental analysis found: C, 69.93%; H, 8.02%; N, 17.05%. Calculated for C_19_H_26_N_4_O (MW 326.44): C, 69.91%; H, 8.03%; N, 17.16%.

***N*-(4-ethylphenyl)-5-(heptylamino)pyrazine-2-carboxamide (5e)**

Cream solid. Yield: 71%. mp 131–132 °C. IR (ATR-Ge, cm^−1^) 3335 (N-H, NH), 3296 (N-H, CONH), 2928, 2856 (C-H, alkyl), 1662 (C=O, CONH), 1592, 1547, 1521 (C=C, Ar). ^1^H NMR (500 MHz, DMSO-*d*_6_) δ 9.98 (s, 1H, CONH), 8.62 (d, *J* = 1.4 Hz, 1H, H3), 7.94 (d, *J* = 1.4 Hz, 1H, H6), 7.81 (t, *J* = 5.5 Hz, 1H, NH), 7.76–7.70 (m, 2H, H2′, H6′), 7.18–7.11 (m, 2H, H3′, H5′), 3.36–3.28 (m, 2H, CH_2_), 2.56 (q, *J* = 7.6 Hz, 2H, CH_2_), 1.54 (h, *J* = 6.6 Hz, 2H, CH_2_), 1.38–1.21 (m, 8H, (CH_2_)_4_), 1.16 (t, *J* = 7.6 Hz, 3H, CH_3_), 0.88–0.82 (m, 3H, CH_3_). ^13^C NMR (126 MHz, DMSO-*d*_6_) δ 162.35, 156.29, 143.01, 138.91, 136.60, 131.93, 131.08, 127.93, 120.17, 40.45, 31.42, 28.71, 28.63, 27.79, 26.63, 22.22, 15.86, 14.10. Elemental analysis found: C, 70.26%; H, 8.36%; N, 16.25%. Calculated for C_20_H_28_N_4_O (MW 340.47): C, 70.56%; H, 8.29%; N, 16.46%

***N*-(4-ethylphenyl)-5-(octylamino)pyrazine-2-carboxamide (5f)**

White solid. Yield: 77%. mp 131–132 °C. IR (ATR-Ge, cm^−1^) 3358 (N-H, NH), 3296 (N-H, CONH), 2928, 2852 (C-H, alkyl), 1662 (C=O, CONH), 1595, 1548, 1523 (C=C, Ar). ^1^H NMR (500 MHz, DMSO-*d*_6_) δ 9.98 (s, 1H, CONH), 8.63 (d, *J* = 1.3 Hz, 1H, H3), 7.94 (d, *J* = 1.3 Hz, 1H, H6), 7.81 (t, *J* = 5.5 Hz, 1H, NH), 7.76–7.70 (m, 2H, H2′, H6′), 7.18–7.11 (m, 2H, , H3′, H5′), 3.36–3.28 (m, 2H, CH_2_), 2.56 (q, *J* = 7.6 Hz, 2H, CH_2_), 1.54 (h, *J* = 6.6 Hz, 2H, CH_2_), 1.30–1.22 (m, 10H, (CH_2_)_5_), 1.16 (t, *J* = 7.6 Hz, 3H, CH_3_), 0.88–0.81 (m, 3H, CH_3_). ^13^C NMR (126 MHz, DMSO-*d*_6_) δ 162.35, 156.30, 143.01, 138.91, 136.60, 131.93, 131.25, 127.93, 120.17, 40.46, 31.41, 28.93, 28.84, 28.70, 27.80, 26.67, 22.25, 15.87, 14.09. Elemental analysis found: C, 71.25%; H, 8.69%; N, 15.74%. Calculated for C_21_H_30_N_4_O (MW 354.50): C, 71.15%; H, 8.53%; N, 15.80%.

***N*-(4-chloro-2-hydroxyphenyl)-5-(propylamino)pyrazine-2-carboxamide (6a)**

Yellow solid. Yield: 44%. mp 248.9–250 °C. IR (ATR-Ge, cm^−1^) 3323 (N-H, CONH, NH), 2965, 2878 (C-H, alkyl), 1664 (C=O, CONH), 1603, 1528 (C=C, Ar). ^1^H NMR (500 MHz, DMSO-*d*_6_) δ 10.75 (s, 1H, CONH), 9.81 (s, 1H, OH), 8.64 (d, *J* = 1.4 Hz, 1H, H3), 8.34 (d, *J* = 8.7 Hz, 1H, H6′), 7.98–7.92 (m, 2H, H6, NH), 6.93 (d, *J* = 2.4 Hz, 1H, H3′), 6.88 (dd, *J* = 8.7, 2.4 Hz, 1H, H5′), 3.35–3.26 (m, 2H, CH_2_), 1.57 (h, *J* = 7.3 Hz, 2H, CH_2_), 0.92 (t, *J* = 7.3 Hz, 3H, CH_3_). ^13^C NMR (126 MHz, DMSO-*d*_6_) δ 161.65, 156.50, 147.46, 142.92, 131.67, 131.13, 126.80, 125.97, 119.91, 119.11, 114.57, 40.33, 21.94, 11.64. Elemental analysis found: C, 54.50%; H, 4.76%; N, 17.99%. Calculated for C_14_H_15_ClN_4_O_2_ (MW 306.75): C, 54.82%; H, 4.93%; N, 18.27%.

**5-(Butylamino)pyrazine-*N*-(4-chloro-2-hydroxyphenyl)-2-carboxamide (6b)**

Pale yellow solid. Yield: 70%. mp 221.8–225.4 °C. IR (ATR-Ge, cm^−1^) 3315 (N-H, CONH, NH), 2930, 2874 (C-H, alkyl), 1663 (C=O, CONH), 1603, 1554, 1526 (C=C, Ar). ^1^H NMR (500 MHz, DMSO-*d*_6_) δ 10.73 (s, 1H, CONH), 9.80 (s, 1H, OH), 8.63 (d, *J* = 1.3 Hz, 1H, H3), 8.34 (d, *J* = 8.7 Hz, 1H, H6′), 7.95–7.89 (m, 2H, H6, NH), 6.93 (d, *J* = 2.3 Hz, 1H, H3′), 6.87 (dd, *J* = 8.7, 2.3 Hz, 1H, H5′), 3.33 (td, *J* = 7.0, 5.4 Hz, 2H, CH_2_), 1.58–1.49 (m, 2H, CH_2_), 1.41–1.30 (m, 2H, CH_2_), 0.90 (t, *J* = 7.0 Hz, 3H, CH_3_). ^13^C NMR (126 MHz, DMSO-*d*_6_) δ 161.65, 156.47, 147.45, 142.93, 131.67, 131.13, 126.79, 125.98, 119.91, 119.11, 114.56, 40.20, 30.76, 19.83, 13.85. Elemental analysis found: C, 56.32%; H, 5.30%; N, 17.55%. Calculated for C_15_H_17_ClN_4_O_2_ (MW 320.78): C, 56.17%; H, 5.34%; N, 17.47%.

***N-*(4-chloro-2-hydroxyphenyl)-5-(pentylamino)pyrazine-2-carboxamide (6c)**

Pale yellow solid. Yield: 23%. mp 206.5–208.7 °C. IR (ATR-Ge, cm^−1^) 3321 (N-H, CONH, NH), 2927, 2872 (C-H, alkyl), 1663 (C=O, CONH), 1604, 1554, 1529 (C=C, Ar). ^1^H NMR (500 MHz, DMSO-*d*_6_) δ 10.77 (s, 1H, CONH), 9.81 (s, 1H, OH), 8.63 (d, *J* = 1.3 Hz, 1H, H3), 8.34 (d, *J* = 8.7 Hz, 1H, H6′), 7.93 (s, 2H, H6, NH), 6.93 (d, *J* = 2.4 Hz, 1H, H3′), 6.87 (dd, *J* = 8.7, 2.4 Hz, 1H, H5′), 3.32 (q, *J* = 6.8 Hz, 2H, CH_2_), 1.55–1.44 (m, 2H, CH_2_), 1.38–1.23 (m, 4H, (CH_2_)_2_), 0.85 (d, *J* = 6.8 Hz, 3H, CH_3_). ^13^C NMR (126 MHz, DMSO-*d*_6_) δ 161.64, 156.47, 147.52, 142.91, 131.75, 131.13, 126.75, 125.97, 119.82, 119.02, 114.59, 40.49, 28.85, 28.33, 22.06, 14.07. Elemental analysis found: C, 56.94%; H, 5.68%; N, 16.48%. Calculated for C_16_H_19_ClN_4_O_2_ (MW 334.8): C, 57.40%; H, 5.72%; N, 16.73%.

***N*-(4-chloro-2-hydroxyphenyl)-5-(hexylamino)pyrazine-2-carboxamide (6d)**

Yellow solid. Yield: 40%. mp 201.7–204.9 °C. IR (ATR-Ge, cm^−1^) 3324 (N-H, CONH, NH), 2929, 2857 (C-H, alkyl), 1664 (C=O, CONH), 1603, 1554, 1527 (C=C, Ar). ^1^H NMR (500 MHz, DMSO-*d*_6_) δ 10.76 (s, 1H, CONH), 9.81 (s, 1H, OH), 8.63 (s, 1H, H3), 8.34 (d, *J* = 8.7 Hz, 1H, H6′), 7.93 (m, 2H, H6, NH), 6.93 (d, *J* = 2.4 Hz, 1H, H3′), 6.87 (dd, *J* = 8.7, 2.4 Hz, 1H, H5′), 3.38–3.24 (m, 2H, CH_2_), 1.54 (p, *J* = 7.1 Hz, 2H, CH_2_), 1.38–1.23 (m, 6H, (CH_2_)_3_), 0.89–0.82 (m, 3H, CH_3_). ^13^C NMR (126 MHz, DMSO-*d*_6_) δ 161.65, 156.46, 147.48, 142.92, 132.41, 131.13, 126.78, 125.98, 119.88, 119.08, 114.56, 40.54, 31.19, 28.62, 26.35, 22.25, 14.08. Elemental analysis found: C, 58.08%; H, 5.60%; N, 16.33%. Calculated for C_17_H_21_ClN_4_O_2_ (MW 348.83): C, 58.53%; H, 6.07%; N, 16.06%.

***N*-(4-chloro-2-hydroxyphenyl)-5-(heptylamino)pyrazine-2-carboxamide (6e)**

Pale yellow solid. Yield: 27%. mp 199.8–200.8 °C. IR (ATR-Ge, cm^−1^) 3317 (N-H, CONH, NH), 2930, 2858 (C-H, alkyl), 1664 (C=O, CONH), 1603, 1527 (C=C, Ar). ^1^H NMR (500 MHz, DMSO-*d*_6_) δ 10.73 (s, 1H, CONH), 9.80 (s, 1H, OH), 8.63 (d, *J* = 1.3 Hz, 1H, H3), 8.34 (d, *J* = 8.7 Hz, 1H, H6′), 7.94–7.89 (m, 2H, H6, NH), 6.92 (d, *J* = 2.4 Hz, 1H, H3′), 6.87 (dd, *J* = 8.7, 2.4 Hz, 1H, H5′), 3.34–3.28 (m, 2H, CH_2_), 1.54 (p, *J* = 7.1 Hz, 2H, CH_2_), 1.37–1.20 (m, 8H, (CH_2_)_4_), 0.84 (t, *J* = 6.8 Hz, 3H, CH_3_). ^13^C NMR (126 MHz, DMSO-*d*_6_) δ 161.65, 156.46, 147.46, 142.92, 131.75, 131.13, 126.79, 125.98, 119.90, 119.10, 114.56, 40.52, 31.43, 28.66, 28.63, 26.64, 22.23, 14.10. Elemental analysis found: C, 59.19%; H, 6.22%; N, 15.07%. Calculated for C_18_H_23_ClN_4_O_2_ (MW 362.86): C, 59.58%; H, 6.39%; N, 15.44%.

***N*-(4-chloro-2-hydroxyphenyl)-5-(octylamino)pyrazine-2-carboxamide (6f)**

Yellow solid. Yield: 15%. mp 196.9–197.9 °C. IR (ATR-Ge, cm^−1^) 3315 (N-H, CONH, NH), 2926, 2855 (C-H, alkyl), 1663 (C=O, CONH), 1603, 1554, 1526 (C=C, Ar). ^1^H NMR (500 MHz, DMSO-*d*_6_) δ 10.74 (s, 1H, CONH), 9.81 (s, 1H, OH), 8.63 (s, 1H, H3), 8.34 (d, *J* = 8.7 Hz, 1H, H6′), 7.93 (s, 2H, NH, H6), 6.92 (d, *J* = 2.4 Hz, 1H, H3′), 6.87 (dd, *J* = 8.7, 2.4 Hz, 1H, H5′), 3.35–3.29 (m, 2H, CH_2_), 1.54 (p, *J* = 7.0 Hz, 2H, CH_2_), 1.32 (dt, *J* = 12.6, 7.0 Hz, 2H, CH_2_), 1.25 (tt, *J* = 12.6, 9.0, 7.7 Hz, 8H, (CH_2_)_4_), 0.84 (t, *J* = 7.0 Hz, 3H, CH_3_). ^13^C NMR (126 MHz, DMSO-*d*_6_) δ 161.64, 156.45, 147.46, 142.92, 131.69, 131.12, 126.78, 125.97, 119.89, 119.09, 114.55, 40.51, 31.40, 28.91, 28.83, 28.63, 26.66, 22.24, 14.09. Elemental analysis found: C, 60.60%; H, 6.71%; N, 14.66%. Calculated for C_19_H_25_ClN_4_O_2_ (MW 376.89): C, 60.55%; H, 6.69%; N, 14.87%.

***N*-(5-chloro-2-hydroxyphenyl)-5-(propylamino)pyrazine-2-carboxamide (7a)**

Pale yellow solid. Yield: 45%. mp 254.0–259.0 °C. IR (ATR-Ge, cm^−1^) 3344 (N-H, NH), 3321 (N-H, CONH), 2934, 2861 (C-H, alkyl), 1672 (C=O, CONH), 1604, 1560, 1530 (C=C, Ar). ^1^H NMR (500 MHz, DMSO-*d*_6_) δ 10.54 (s, 1H, CONH), 9.86 (s, 1H, OH), 8.64 (d, *J* = 1.4 Hz, 1H, H3), 8.41 (d, *J* = 2.6 Hz, 1H, H6′), 7.98 (t, *J* = 5.7 Hz, 1H, NH), 7.94 (d, *J* = 1.4 Hz, 1H, H6), 7.01–6.88 (m, 2H, H3′, H4′), 3.30 (td, *J* = 7.2, 5.7 Hz, 2H, CH_2_), 1.57 (h, *J* = 7.2 Hz, 2H, CH_2_), 0.92 (t, *J* = 7.2 Hz, 3H, CH_3_). ^13^C NMR (126 MHz, DMSO-*d*_6_) δ 161.83, 156.54, 145.25, 143.04, 131.72, 130.98, 127.94, 122.93, 122.76, 118.31, 115.77, 42.34, 21.93, 11.64. Elemental analysis found: C, 54.62%; H, 4.79%; N, 17.91%. Calculated for C_14_H_15_ClN_4_O_2_ (MW 306.75): C, 54.82%; H, 4.93%; N, 18.27%.

**5-(Butylamino)pyrazine-*N*-(5-chloro-2-hydroxyphenyl)-2-carboxamide (7b)**

Pale yellow solid. Yield: 40%. mp 257.9–262.1 °C. IR (ATR-Ge, cm^−1^) 3329 (N-H, CONH, NH), 2925, 2861 (C-H, alkyl), 1668 (C=O, CONH), 1600, 1558, 1528 (C=C, Ar). ^1^H NMR (500 MHz, DMSO-*d*_6_) δ 10.54 (s, 1H, CONH), 9.85 (s, 1H, OH), 8.64 (d, *J* = 1.3 Hz, 1H, H3), 8.41 (d, *J* = 2.5 Hz, 1H, H6′), 7.95 (t, *J* = 5.6 Hz, 1H, NH), 7.93 (d, *J* = 1.3 Hz, 1H, H6), 6.95 (dd, *J* = 8.6, 2.5 Hz, 1H, H4′), 6.91 (d, *J* = 8.6 Hz, 1H, H3′), 3.36–3,30 (m, 2H, CH_2_), 1.58–1.49 (m, 2H, CH_2_), 1.41–1.30 (m, 2H, CH_2_), 0.90 (t, *J* = 7.3 Hz, 3H, CH_3_). ^13^C NMR (126 MHz, DMSO-*d*_6_) δ 161.82, 156.51, 145.22, 143.05, 131.87, 130.95, 127.93, 122.92, 122.77, 118.30, 115.77, 40.52, 30.74, 19.83, 13.85. Elemental analysis found: C, 55.90%; H, 5.18%; N, 17.45%. Calculated for C_15_H_17_ClN_4_O_2_ (MW 320.78): C, 56.17%; H, 5.34%; N, 17.47%.

***N*-(5-chloro-2-hydroxyphenyl)-5-(pentylamino)pyrazine-2-carboxamide (7c)**

Cream solid. Yield: 55%. mp 250.3–255.1 °C. IR (ATR-Ge, cm^−1^) 3328 (N-H, CONH, NH), 2929, 2861 (C-H, alkyl), 1667 (C=O, CONH), 1600, 1558, 1528 (C=C, Ar). ^1^H NMR (500 MHz, DMSO-*d*_6_) δ 10.54 (s, 1H, CONH), 9.86 (s, 1H, OH), 8.64 (d, *J* = 1.4 Hz, 1H, H3), 8.41 (d, *J* = 2.5 Hz, 1H, H6′), 7.95 (t, *J* = 5.5 Hz, 1H, NH), 7.93 (d, *J* = 1.4 Hz, 1H, H6), 6.95 (dd, *J* = 8.5, 2.5 Hz, 1H, H4′), 6.91 (d, *J* = 8.5 Hz, 1H, H3′), 3.36–3.30 (m, 2H, CH_2_), 1.55 (p, *J* = 7.1 Hz, 2H, CH_2_), 1.31 (h, *J* = 7,1, 4.6, Hz, 4H, (CH_2_)_2_), 0.90–0.84 (m, 3H, CH_3_). ^13^C NMR (126 MHz, DMSO-*d*_6_) δ 161.82, 156.50, 145.22, 143.05, 131.82, 130.95, 127.93, 122.92, 122.77, 118.30, 115.76, 40.50, 28.85, 28.32, 22.06, 14.07. Elemental analysis found: C, 57.18%; H, 5.64%; N, 16.79%. Calculated for C_16_H_19_ClN_4_O_2_ (MW 334.83): C, 57.40%; H, 5.72%; N, 16.73%

***N*-(5-chloro-2-hydroxyphenyl)-5-(hexylamino)pyrazine-2-carboxamide (7d)**

Pale yellow solid. Yield: 53%. mp 249.3–251.9 °C. IR (ATR-Ge, cm^−1^) 3329 (N-H, CONH, NH), 2929, 2858 (C-H, alkyl), 1668 (C=O, CONH), 1600, 1558, 1529 (C=C, Ar). ^1^H NMR (500 MHz, DMSO-*d*_6_) δ 10.16 (s, 1H, CONH), 9.87 (s, 1H, OH), 8.64 (d, *J* = 1.4 Hz, 1H, H3), 8.41 (d, *J* = 2.5 Hz, 1H, H6′), 7.96 (t, *J* = 5.7 Hz, 1H, NH), 7.93 (*J* = 1.4 Hz, 1H, H6), 6.97–6.88 (m, 2H, H3′, H4′) 3.35–3.26 (m, 2H, CH_2_), 1.54 (p, *J* = 7.2 Hz, 2H, CH_2_), 1.36–1.30 (m, 2H, CH_2_), 1.32–1.21 (m, 4H, (CH_2_)_2_), 0.89–0.81 (m, 3H, CH_3_). ^13^C NMR (126 MHz, DMSO-*d*_6_) δ 162.36, 156.51, 145.32, 143.07, 131.91, 131.06, 128.04, 123.08, 122.77, 118.32, 115.85, 40.55, 31.51, 28.78, 26.79, 22.27, 14.10. Elemental analysis found: C, 58.05%; H, 5.91%; N, 15.95%. Calculated for C_17_H_21_ClN_4_O_2_ (MW 348.83): C, 58.53%; H, 6.07%; N, 16.06%.

***N*-(5-chloro-2-hydroxyphenyl)-5-(heptylamino)pyrazine-2-carboxamide (7e)**

Cream solid. Yield: 34%. mp 243.6–246.0 °C. IR (ATR-Ge, cm^−1^) 3354 (N-H, NH), 3331 (N-H, CONH), 2927, 2856 (C-H, alkyl), 1668 (C=O, CONH), 1600, 1557, 1529 (C=C, Ar). ^1^H NMR (500 MHz, DMSO-*d*_6_) δ 10.56 (s, 1H, CONH), 9.85 (s, 1H, OH), 8.61 (d, *J* = 1.4 Hz ,1H, H3), 8.38 (d, *J* = 2.6 Hz, 1H, H6′), 7.92 (d, *J* = 5.6 Hz, 1H, NH), 7.91 (d, *J* = 1.4 Hz, 1H, H6), 6.97–6.88 (m, 2H, H3′, H4′), 3.29 (t, *J* = 7.1 Hz, 2H, CH_2_), 1.52 (p, *J* = 7.1 Hz, 2H, CH_2_), 1.30 (d, *J* = 7.1 Hz, 2H, CH_2_), 1.32–1.21 (m, 6H, (CH_2_)_3_), 0.82 (t, *J* = 6.8 Hz, 3H, CH_3_). ^13^C NMR (126 MHz, DMSO-*d*_6_) δ 162.08, 156.66, 145.46, 143.21, 131.77, 131.09, 128.08, 123.18, 122.97, 118.52, 115.95, 40.74, 31.60, 28.80, 28.58, 26.80, 22.41, 14.28. Elemental analysis found: C, 59.14%; H, 6.22%; N, 15.21%. Calculated for C_18_H_23_ClN_4_O_2_ (MW 362.86): C, 59.58%; H, 6.39%; N, 15.44%.

***N*-(5-chloro-2-hydroxyphenyl)-5-(octylamino)pyrazine-2-carboxamide (7f)**

Pale yellow solid. Yield: 25%. mp 239.7–242.4 °C. IR (ATR-Ge, cm^−1^) 3362 (N-H, NH), 3330 (N-H, CONH), 2928, 2856 (C-H, alkyl), 1669 (C=O, CONH), 1600, 1558, 1529 (C=C, Ar). ^1^H NMR (500 MHz, DMSO-*d*_6_) δ 10.55 (s, 1H, CONH), 9.86 (s, 1H, OH), 8.64 (d, *J* = 1.4 Hz, 1H, H3), 8.41 (d, *J* = 2.4 Hz, 1H, H6′), 7.96 (d, *J* = 5.9 Hz, 1H, NH), 7.93 (d, *J* = 1.4 Hz, 1H, H6), 6.97–6.89 (m, 2H, H3′, H4′), 3.35–3.29 (m, 2H, CH_2_), 1.54 (p, *J* = 7.1 Hz, 2H, CH_2_), 1.37–1.21 (m, 10H, (CH_2_)_5_), 0.84 (t, *J* = 6.6 Hz, 3H, CH_3_). ^13^C NMR (126 MHz, DMSO-*d*_6_) δ 161.82, 156.50, 145.28, 143.04, 133.82, 130.96, 127.94, 122.90, 122.72, 118.28, 115.76, 40.52, 31.41, 28.92, 28.84, 28.63, 26.67, 22.25, 14.09. Elemental analysis found: C, 60.17%; H, 6.51%; N, 14.62%. Calculated for C_19_H_25_ClN_4_O_2_ (MW 376.89): C, 60.55%; H, 6.69%; N, 14.87%.

### 3.4. Biological Methods

#### 3.4.1. Antimycobacterial In Vitro Activity Screening Against *Mycobacterium tuberculosis* H37Ra, *Mycobacterium smegmatis*, and *Mycobacterium aurum*

Antimycobacterial assays were performed with fast growing *Mycobacterium smegmatis* DSM 43465 (ATCC 607), *Mycobacterium aurum* DSM 43999 (ATCC 23366) from German Collection of Microorganisms and Cell Cultures (Braunschweig, Germany) and with an avirulent strain of *Mycobacterium tuberculosis* H37Ra ITM-M006710 (ATCC 9431) from Belgian Co-ordinated Collections of Micro-organisms. The technique used for activity determination was microdilution broth panel method using 96-well microtitration plates. Culturing medium was Middlebrook 7H9 broth (Sigma–Aldrich, Steinheim, Germany) enriched with 0.4% of glycerol (Sigma–Aldrich) and 10% of Middlebrook OADC growth supplement (Himedia, Mumbai, India).

Mycobacterial strains were cultured on Middlebrook 7H9 agar and suspensions were prepared in Middlebrook 7H9 broth. Final density was adjusted to value 1.0 according to McFarland scale and diluted in ratio 1:20 (for fast growing mycobacteria) or 1:10 (for *M. tbc*) with broth.

Tested compounds were dissolved in DMSO (Sigma–Aldrich) then Middlebrook broth was added to obtain concentration 2000 µg/mL. Standards used for activity determination were isoniazid (INH), rifampicin (RIF) and ciprofloxacin (CPX) (Sigma–Aldrich). Final concentrations were reached by binary dilution and addition of mycobacterial suspension and were set as 500, 250, 125, 62.5, 31.25, 15.625, 7.81, 3.91, 1.56, and 0.78 µg/mL. Isoniazid was diluted in range 500–3.91 µg/mL for screening against fast growing mycobacteria and in range 1–0.0078 µg/mL for screening against *M. tuberculosis*. Rifampicin final concentrations ranged from 50 to 0.39 µg/mL for fast growing mycobacteria and from 1 to 0.0078 µg/mL for *M. tuberculosis* H37Ra. Ciprofloxacin was used for screening antimycobacterial activity with the final concentrations 1, 0.5, 0.25, 0.125, 0.0625, 0.0313, 0.0156, and 0.0078 µg/mL. The final concentration of DMSO did neither exceeded 2.5% (*v*/*v*) and did not affect the growth of *M. smegmatis, M. aurum* nor *M. tuberculosis*. Positive (broth, DMSO, bacteria) and negative (broth, DMSO) controls were included.

Plates were sealed with polyester adhesive film and incubated in dark at 37 °C without agitation. The addition of 0.01% solution of resazurin sodium salt followed after 48 h of incubation for *M. smegmatis*, after 72 h of incubation for *M. aurum* and after 120 h of incubation for *M. tuberculosis*, respectively. Stain was prepared by dissolving resazurin sodium salt (Sigma–Aldrich) in deionized water to get 0.02% solution. Then 10% aqueous solution of Tween 80 (Sigma–Aldrich) was prepared. Both liquids were mixed up making use of the same volumes and filtered through syringe membrane filter. Microtitration panels were then incubated for further 2.5 h for determination of activity against *M. smegmatis*, 4 h for *M. aurum* and 24 h for *M. tuberculosis*, respectively.

Antimycobacterial activity was expressed as minimal inhibition concentration (MIC) and the value was read based on stain color change (blue color—active compound; pink color—not active compound). All experiments were conducted in duplicate.

#### 3.4.2. Antimycobacterial In Vitro Activity Screening Against *Mycobacterium kansasii* and *Mycobacterium avium*

A 96 well plate microdilution broth method was performed. Tested *M. kansasii* CNCTC My 235/80 (ATCC 12478) and *M. avium* ssp. *avium* CNCTC My 80/72 (ATCC 15769) were obtained from the Czech National Collection of Type Cultures (CNCTC), National Institute of Public Health (Prague, Czech Republic). Middlebrook 7H9 broth of declared pH = 6.6 (Sigma–Aldrich) enriched with 0.4% of glycerol (Sigma–Aldrich) and 10% of OADC growth supplement (oleic acid, albumin, dextrose, catalase; Himedia, Mumbai, India) was used for cultivation. Tested compounds were dissolved and diluted in DMSO and mixed with broth (25 µL of DMSO solution in 4.475 mL of broth) and placed (100 µL) into microplate wells. Mycobacterial inocula were suspended in isotonic saline solution and the density was adjusted to 0.5–1.0 according to McFarland scale. These suspensions were diluted by 10^−1^ and used to inoculate the testing wells, adding 100 µL of mycobacterial suspension per well. Final concentrations of tested compounds in wells were 100, 50, 25, 12.5, 6.25, 3.13, and 1.56 µg/mL. The INH was used as positive control (inhibition of growth). Negative control (visible growth) consisted of broth plus mycobacterial suspension plus DMSO (purity of broth). A total of 30 µL of Alamar Blue working solution (1:1 mixture of 0.01% resazurin sodium salt (aq. sol.) and 10% Tween 80) was added after five days of incubation. Results were then determined after 24 h of incubation. The MIC (in µg/mL) was determined as the lowest concentration that prevented the blue to pink color change. All experiments were conducted in duplicates.

#### 3.4.3. HepG2 Cytotoxicity Determination

The human hepatocellular liver carcinoma cell line HepG2 purchased from Health Protection Agency Culture Collections (ECACC, Salisbury, UK) was cultured in MEM (Minimum Essentials Eagle Medium, Sigma–Aldrich) supplemented with 10% fetal bovine serum (PAA Laboratories, Pasching, Austria), 1% L-glutamine solution (Sigma–Aldrich) and non-essential amino acid solution (Sigma–Aldrich) in a humidified atmosphere containing 5% CO_2_ at 37 °C.

For subculturing, the cells were harvested after trypsin/EDTA (Sigma–Aldrich) treatment at 37 °C. To evaluate cytotoxicity, the cells treated with the tested substances were used as experimental groups whereas untreated HepG2 cells served as controls.

The cells were seeded in density 10,000 cells per well in a 96 well plate. The next day the cells were treated with each of the tested substances dissolved in DMSO. The tested substances were prepared at different incubation concentrations in triplicates according to their solubility. Simultaneously, the controls representing 100% cell viability, 0% cell viability (the cells treated with 10% DMSO), no cell control and vehiculum controls were also prepared in triplicates. After 24 h incubation in a humidified atmosphere containing 5% CO_2_ at 37%, the reagent from the kit CellTiter 96 AQueous One Solution Cell Proliferation Assay (CellTiter 96; PROMEGA, Fitchburg, USA) was added. After 2h incubation at 37% absorbance of samples was recorded at 490 nm (TECAN, Infinita M200, Austria). A standard toxicological parameter IC_50_ was calculated by nonlinear regression from a semilogarithmic plot of incubation concentration versus percentage of absorbance relative to untreated controls using GraphPad Prism 8 software.

## 4. Conclusions

The present work reflects the successful design and synthesis of novel, potentially active antimycobacterial agents structurally based on pyrazinamide. A library of forty-two compounds was obtained. Final compounds were evaluated for in vitro growth inhibition activity against *Mycobacterium tuberculosis* H37Ra and several non-tuberculous mycobacteria. As a complementary test, the final compounds were evaluated in a panel of clinically important Gram-positive and Gram-negative bacteria and fungal pathogens. No antibacterial and sporadic antifungal activity has been found, but some of the compounds proved the same or even better in vitro antimycobacterial activity in comparison with known antimycobacterial drugs.

From the structure–activity relationship point of view, we explored the chemical space around previously evaluated antimycobacterial 5-alkylamino-*N*-phenylpyrazine-2-carboxamides [14] to find out that the benzene ring tolerates different substitution patterns, including lipo/hydrophilic and electron donating/withdrawing substituents.

Compounds with relatively good selectivity index (calculated for *Mtb* H37Ra and cytotoxicity on HepG2 liver cancer cells) need to be highlighted: *N*-(4-hydroxyphenyl)-5-(pentylamino)pyrazine-2-carboxamide (**3c**, MIC = 3.91 µg/mL, 13.02 µM, SI >38), 5-(heptylamino)-*N*-(*p*-tolyl)pyrazine-2-carboxamide (**4e**, MIC = 0.78 µg/mL, 2.39 µM, SI >20), *N*-(4-ethylphenyl)-5-(propylamino)pyrazine-2-carboxamide (**5a**, MIC = 7.81 µg/mL, 27.47 µM, SI = 18), 5-(butylamino)-*N*-(3-(trifluoromethyl)phenyl)pyrazine-2-carboxamide (**1b**, MIC = 3.13 µg/mL, 9.24 µM, SI = 14) and 5-(hexylamino)-*N*-(*p*-tolyl)pyrazine-2-carboxamide (**4d**, MIC = 1.56 µg/mL, 4.99 µM, SI > 10).

Out of the derivatives mentioned in the previous paragraph, compounds **1b** and **3d** exerted a broad spectrum of antimycobacterial activity, inhibiting the growth of at least three out of five tested strains. We propose derivative **4e** (for its potency against *Mtb* H37Ra and good SI) and derivatives **1b** and **3d** (for their broad spectrum of antimycobacterial activity and favorable SI) as starting points for further development.

## Figures and Tables

**Table 1:** In vitro antimycobacterial activity against *Mtb* H37Ra, *Mycobacterium aurum* and *M. smegmatis* (MIC in µg/mL), cytotoxicity (IC_50_), selectivity index (SI), and calculated lipophilicity (log*P*) of prepared compounds.

No.	R^1^	R^2^	*Mtb* H37Ra	*Mycobacterium* *aurum*	*Mycobacterium* *smegmatis*	HepG2 IC_50_ (µM)	SI ^a^	log*P*
1	3-CF_3_	-	7.81	≥500	≥500	123.8	4.78	2.41
1a	3-CF_3_	Propyl	6.25	≥500	≥500	>25	>1.3	2.55
1b	3-CF_3_	Butyl	3.13	6.25	3.13	125.6	13.6	2.96
1c	3-CF_3_	Pentyl	3.13	3.13	1.56	18.9	2.13	3.38
1d	3-CF_3_	Hexyl	≥500	25	≥500	>25	n.a.	3.80
1e	3-CF_3_	Heptyl	≥500	62.5	≥500	222.9	n.a.	4.22
1f	3-CF_3_	Octyl	≥500	≥500	≥500	66.2	n.a.	4.63
2	3-OH	-	31.25	≥500	≥500	>500	>3.99	1.10
2a	3-OH	Propyl	7.81	≥500	≥500	>100	>3.5	1.24
2b	3-OH	Butyl	7.81	≥500	≥500	>25	>0.92	1.65
2c	3-OH	Pentyl	≥500	≥500	≥500	>10	n.a.	2.07
2d	3-OH	Hexyl	≥500	≥500	≥500	>10	n.a.	2.49
2e	3-OH	Heptyl	≥500	≥500	250	>10	n.a.	2.91
2f	3-OH	Octyl	≥500	≥500	250	>50	n.a.	3.32
3	4-OH	-	62.50	≥250	≥250	350.2	1.40	1.10
3a	4-OH	Propyl	15.63	≥500	≥500	410.5	7.18	1.24
3b	4-OH	Butyl	31.25	≥500	≥500	377.9	3.46	1.65
3c	4-OH	Pentyl	3.91	≥500	31.25	>500	>38.4	2.07
3d	4-OH	Hexyl	3.91	≥500	250	58.6	4.71	2.49
3e	4-OH	Heptyl	3.91	≥500	31.25	44.8	3.76	2.91
3f	4-OH	Octyl	3.13	≥250	≥250	60.2	6.6	3.32
4	4-CH_3_	-	≥500	≥500	≥500	>50	n.a.	1.98
4a	4-CH_3_	Propyl	15.63	≥500	31.25	>100	>1.73	2.11
4b	4-CH_3_	Butyl	≥ 250	≥250	≥250	>50	n.a.	2.53
4c	4-CH_3_	Pentyl	≥500	≥500	≥500	>25	n.a.	2.95
4d	4-CH_3_	Hexyl	1.56	≥500	≥500	>50	>10.01	3.36
4e	4-CH_3_	Heptyl	0.78	≥500	≥500	>50	>20.91	3.78
4f	4-CH_3_	Octyl	≥250	≥250	≥250	>25	n.a.	4.20
5	4-C_2_H_5_	-	250	≥500	≥500	>50	n.a.	3.29
5a	4-C_2_H_5_	Propyl	7.81	≥500	7.81	494.6	18.01	2.53
5b	4-C_2_H_5_	Butyl	≥500	≥500	≥500	>50	n.a.	2.95
5c	4-C_2_H_5_	Pentyl	≥500	≥500	≥500	>25	n.a.	3.36
5d	4-C_2_H_5_	Hexyl	≥500	≥500	≥500	>25	n.a.	3.78
5e	4-C_2_H_5_	Heptyl	≥500	≥500	≥500	>25	n.a.	4.20
5f	4-C_2_H_5_	Octyl	≥250	≥250	≥250	>10	n.a.	4.62
6	4-Cl-2-OH	-	≥500	≥500	≥500	>250	n.a.	1.66
6a	4-Cl-2-OH	Propyl	≥500	≥500	3.91	n.a.	n.a.	1.79
6b	4-Cl-2-OH	Butyl	≥500	≥500	3.91	>50	n.a.	2.21
6c	4-Cl-2-OH	Pentyl	≥500	≥500	3.91	81.1	n.a.	2.63
6d	4-Cl-2-OH	Hexyl	3.13	≥500	3.91	>50	>5.27	3.05
6e	4-Cl-2-OH	Heptyl	≥500	≥500	≥500	>25	n.a.	3.46
6f	4-Cl-2-OH	Octyl	≥500	≥500	7.81	>50	n.a.	3.88
7	5-Cl-2-OH	-	15.63	31.25	15.63	151.2	2.75	1.66
7a	5-Cl-2-OH	Propyl	≥500	250	<3.91	>25	n.a.	1.79
7b	5-Cl-2-OH	Butyl	≥500	≥500	3.91	>25	n.a.	2.21
7c	5-Cl-2-OH	Pentyl	≥500	≥500	≥500	>25	n.a.	2.63
7d	5-Cl-2-OH	Hexyl	≥500	≥500	≥500	>25	n.a.	3.05
7e	5-Cl-2-OH	Heptyl	≥500	≥500	31.25	>10	>0.32	3.46
7f	5-Cl-2-OH	Octyl	≥500	≥500	250	>50	n.a.	3.88
INH			0.125–0.250	3.91–7.81	7.81–15.63			
RIF			0.0039–0.0078	0.39–0.78	6.25–12.5			
CPX			0.125–0.25	0.0156–0.0313	0.0625–0.125			

^a^ SI—Selectivity index, values calculated from MIC against *Mtb* H37Ra as IC_50_/MIC (in µM); n.a.—not available; INH—isoniazid; RIF—rifampicin; CPX—ciprofloxacin. In the column HepG2 IC_50_ (µM), symbol “>” means that measurement at higher concentration was not reproducible due to the precipitation of the tested compound in the cell culture medium.

**Table 2:** In vitro antimycobacterial activity (expressed as MIC in µg/mL) against non-tuberculous mycobacterial strains of *M. kansasii* and *M. avium*.^a^

No.	R^1^	R^2^	*Mycobacterium* *kansasii*	*Mycobacterium* *avium*
3c	4-OH	Pentyl	12.5	50
3f	4-OH	Octyl	6.25	>100
4d	4-CH_3_	Hexyl	12.5	>100
4e	4-CH_3_	Heptyl	12.5	>100
6c	4-Cl-2-OH	Pentyl	25	>100
6d	4-Cl-2-OH	Hexyl	25	>100
6e	4-Cl-2-OH	Heptyl	25	>100
INH			25	25

^a^ Compounds **1**, **1a**, **1c**, **1f**, **2**, **3**, **3d**, **3e**, **4**, **5**, **5f**, **6**, **6f**, and **7** were not tested in this study. Other compounds not mentioned in the table were inactive against both strains (MIC > 100 µg/mL).

**Table 3:** Significant anti-candidal activity of final compounds expressed as MIC in µM.

No.	R^1^	R^2^	CA	CK	CP
1c	3-CF_3_	Pentyl	>125	62.5	125
2a	3-OH	Propyl	>125	62.5	>125
3b	4-OH	Butyl	62.5	>250	250
3c	4-OH	Pentyl	62.5	>125	>125
4d	4-CH_3_	Hexyl	>125	125	125
Amphotericin B			0.5 µg/mL (0.54 µM)	1 µg/mL (1.08 µM)	0.5 µg/mL (0.54 µM)

CA—*Candida albicans*; CK—*Candida krusei*; CP—*Candida parapsilosis*.

## Supplementary Materials

Representative ^1^H and ^13^C NMR spectra of selected compounds (all spectra in the form of FID files can be retrieved from the corresponding author J.Z. upon request); methodology and full results of antibacterial testing; methodology and full results of antifungal testing are available online.
