# Supplementary material for: 5-Alkylamino-N-phenylpyrazine-2-carboxamides: Design, Preparation, and Antimycobacterial Evaluation
Source: Molecules. 2020 Mar 28;25(7):1561. doi: 10.3390/molecules25071561 (PMC7180572; doi:10.3390/molecules25071561)
Supplement: Supplementary file 1 [file molecules-25-01561-s001.pdf]

5-Alkylamino-*N*-phenylpyrazine-2-carboxamides: Design, Preparation and Antimycobacterial Evaluation

Weronika Ambrożkiewicz [1,*], Marta Kučerová-Chlupáčová [1], Ondřej Janďourek [1], Klára Konečná [1], Pavla Paterová [2], Pavel Bárta [1], Jarmila Vinšová [1], Martin Doležal [1] and Jan Zitko [1,*]

[1] Faculty of Pharmacy in Hradec Králové, Charles University, Heyrovského 1203, 500 05 Hradec Králové, Czech Republic; kucerom@faf.cuni.cz (M.K.); jando6aa@faf.cuni.cz (O.J.); konecna@faf.cuni.cz (K.K.); bartp7aa@faf.cuni.cz (P.B.); vinsova@faf.cuni.cz (J.V.); dolezalm@faf.cuni.cz (M.D.)

[2] Department of Clinical Microbiology, Faculty Hospital, Sokolská 581, 500 05 Hradec Králové, Czech Republic; pavla.paterova@fnhk.cz

* Correspondence: weronika.ambrozkiewicz@gmail.com (W.A.); jan.zitko@faf.cuni.cz (J.Z.); Tel.: +420-495-067-409 (J.Z.); Fax: +420-495-518-002 (J.Z.)

Supplementary Material

Table of Contents

Methodology 2

Evaluation of In *Vitro Antibacterial* Activity 2

Evaluation of *In Vitro* Antifungal Activity 2

Results 3

^{1}H and ^{13}C NMR spectra of compound 1b 3

^{1}H and ^{13}C NMR spectra of compound 1f 4

^{1}H and ^{13}C NMR spectra of compound 2a 5

^{1}H and ^{13}C NMR spectra of compound 2d 6

^{1}H and ^{13}C NMR spectra of compound 3b 7

^{1}H and ^{13}C NMR spectra of compound 3f 8

^{1}H and ^{13}C NMR spectra of compound 4a 9

^{1}H and ^{13}C NMR spectra of compound 4d 10

^{1}H and ^{13}C NMR spectra of compound 5a 11

^{1}H and ^{13}C NMR spectra of compound 5e 12

Full results of antibacterial activity 13

Full results of antifungal activity 16

References 18

Methodology

Evaluation of In *Vitro Antibacterial* Activity

Microdilution broth method [1]. Tested strains were from the Czech Collection of Microorganisms (CCM, Brno, Czech Republic). All strains were subcultured on Mueller-Hinton agar (MHA) (Difco/Becton Dickinson, Detroit, MI, USA) at 35 °C and maintained on the same medium at 4 °C. The compounds were dissolved in DMSO, and the antibacterial activity was determined in cation adjusted Mueller-Hinton liquid broth (Difco/Becton Dickinson) buffered to pH 7.0. Positive controls consisted of test microbe solely, while negative controls consisted of cultivation medium and DMSO. The final concentration of DMSO in the testing medium did not exceed 1% (v/v) of the total solution composition. MIC was determined after 24 and 48 h of static incubation at 35 °C by visual inspection or using Alamar Blue dye. The standards were **gentamicin** [MIC against *Staphylococcus aureus* 1 µg/mL (48 h); *Staphylococcus aureus* methicillin resistant 16–32 µg/mL (48 h); *Enterococcus faecalis* 8 µg/mL (48 h); *Escherichia coli* 1–2 µg/mL (48 h); *Pseudomonas aeruginosa* 0.5 µg/mL (48 h); *Staphylococcus epidermidis* >8 µg/mL (48 h); *Klebsiella pneumoniae* >8 µg/mL (48 h); *Serratia marcescens* 2 µg/mL (48 h)] and **ciprofloxacin** [MIC against *Staphylococcus aureus* 0.128–0.256 µg/mL (48 h); *Staphylococcus aureus* methicillin resistant 0.128 µg/mL (48 h); *Enterococcus faecalis* 0.512 µg/mL (48 h); *Escherichia coli* 0.008 µg/mL (48 h); *Pseudomonas aeruginosa* 0.128 µg/mL (48 h); *Staphylococcus epidermidis* >1.024 µg/mL (48 h); *Klebsiella pneumoniae* >1.024 µg/mL (48 h); *Serratia marcescens* 0.256 µg/mL (48 h)]. All experiments were conducted in duplicates. For the results to be valid, the difference in MIC for one compound determined from two parallel measurements must not be greater than one step on the dilution scale.

Evaluation of *In Vitro* Antifungal Activity

Microdilution broth method [2, 3]. Tested strains were from the Czech Collection of Microorganisms (CCM). Compounds were dissolved in DMSO and diluted in a twofold manner with RPMI 1640 medium, with glutamine and 2% glucose, buffered to pH 7.0 with MOPS (3-morpholinopropane-1-sulfonic acid). The final concentration of DMSO in the testing medium did not exceed 1% (v/v) of the total solution composition. Static incubation was performed in the dark and in humid atmosphere, at 35 °C, for 24 and 48 h (72 and 120 h for Trichophyton interdigitale). Positive controls consisted of test microbe solely, while negative controls consisted of cultivation medium and DMSO. MIC was inspected visually or based on Alamar Blue indication. The standards were **amphotericin B** [MIC against *Candida albicans* 0.5 µg/mL (48 h); *C. krusei* 1 µg/mL (48 h); *C. parapsilosis* 0.5 µg/mL (48 h); *C. tropicalis* 1 µg/mL (48 h); *Aspergillus flavus* 8 µg/mL (48 h); *Lichtheimia corymbifera* 0.5 µg/mL (48 h); *Trichophyton interdigitale* 2 µg/mL (72 h); *Aspergillus fumigatus* 1 µg/mL (48 h)] and **voriconazole** [MIC against *Candida albicans* >16 µg/mL (48 h); *C. krusei* 0.5 µg/mL (48 h); *C. parapsilosis* 8 µg/mL (48 h); *C. tropicalis* >16 µg/mL (48 h); *Aspergillus flavus* >16 µg/mL (48 h); *Lichtheimia corymbifera* >16 µg/mL (48 h*); Trichophyton interdigitale* >16 µg/mL (72 h); *Aspergillus fumigatus* 1 µg/mL (48 h)]. All experiments were conducted in duplicates. For the results to be valid, the difference in MIC for one compound determined from two parallel measurements must not be greater than one step on the dilution scale.

Results

^{1}H and ^{13}C NMR spectra of compound 1b

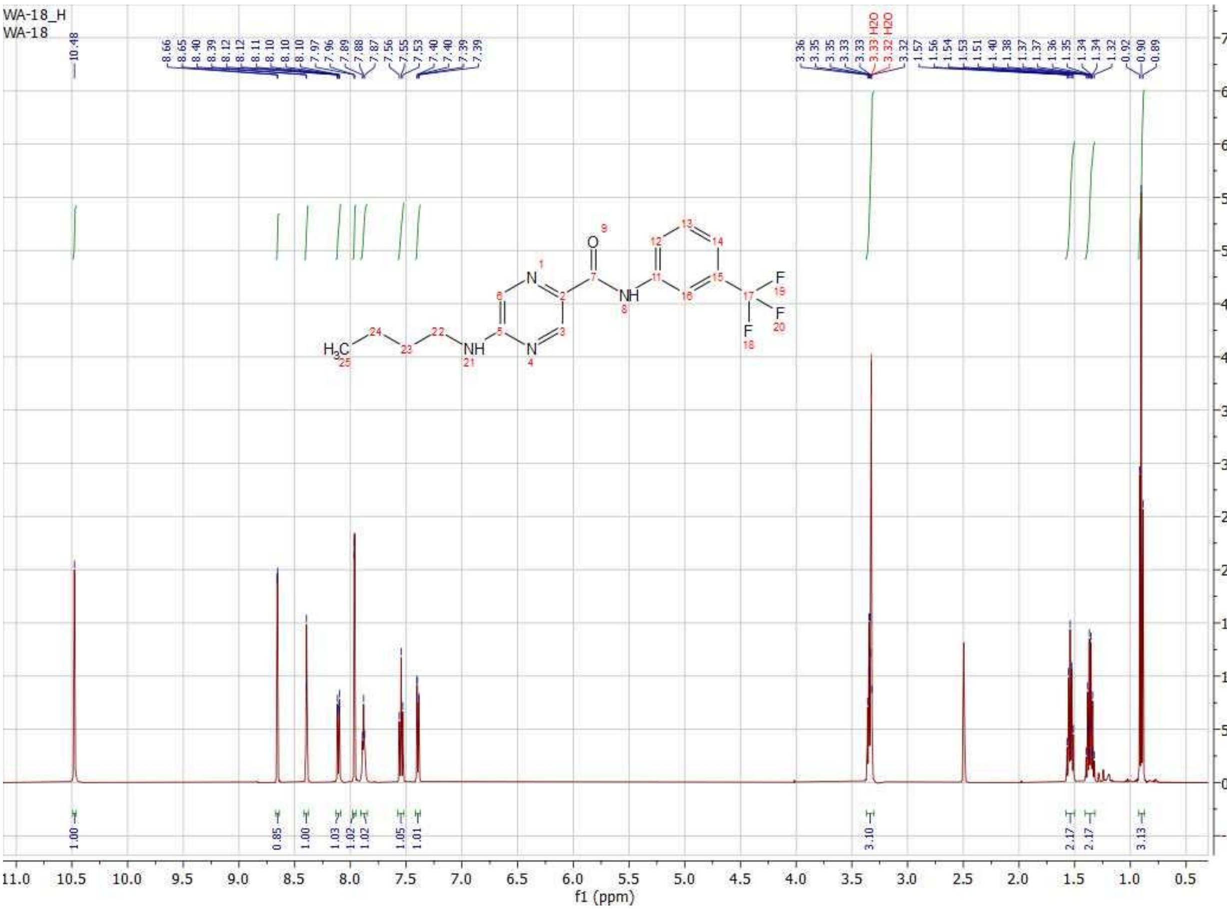

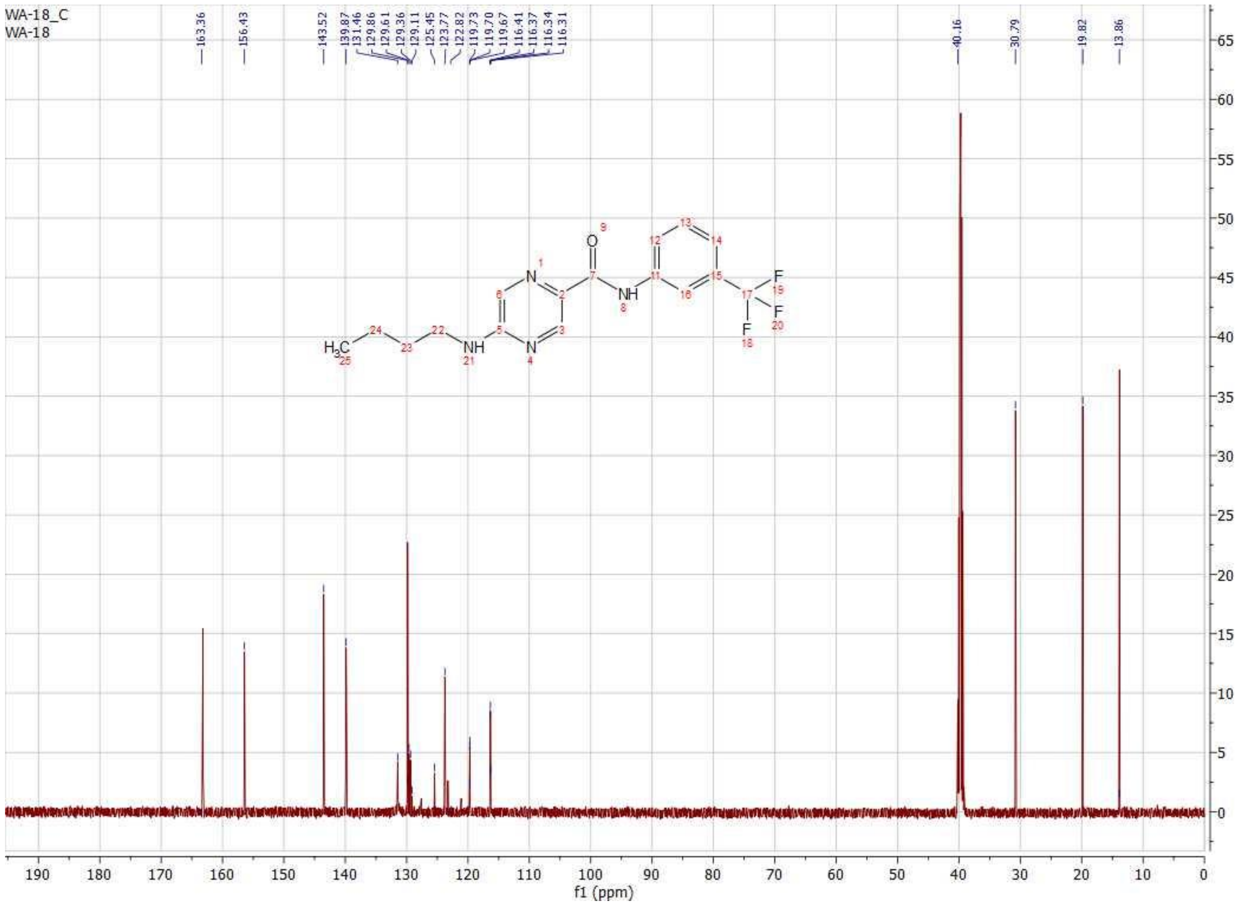

^{1}H and ^{13}C NMR spectra of compound 1f

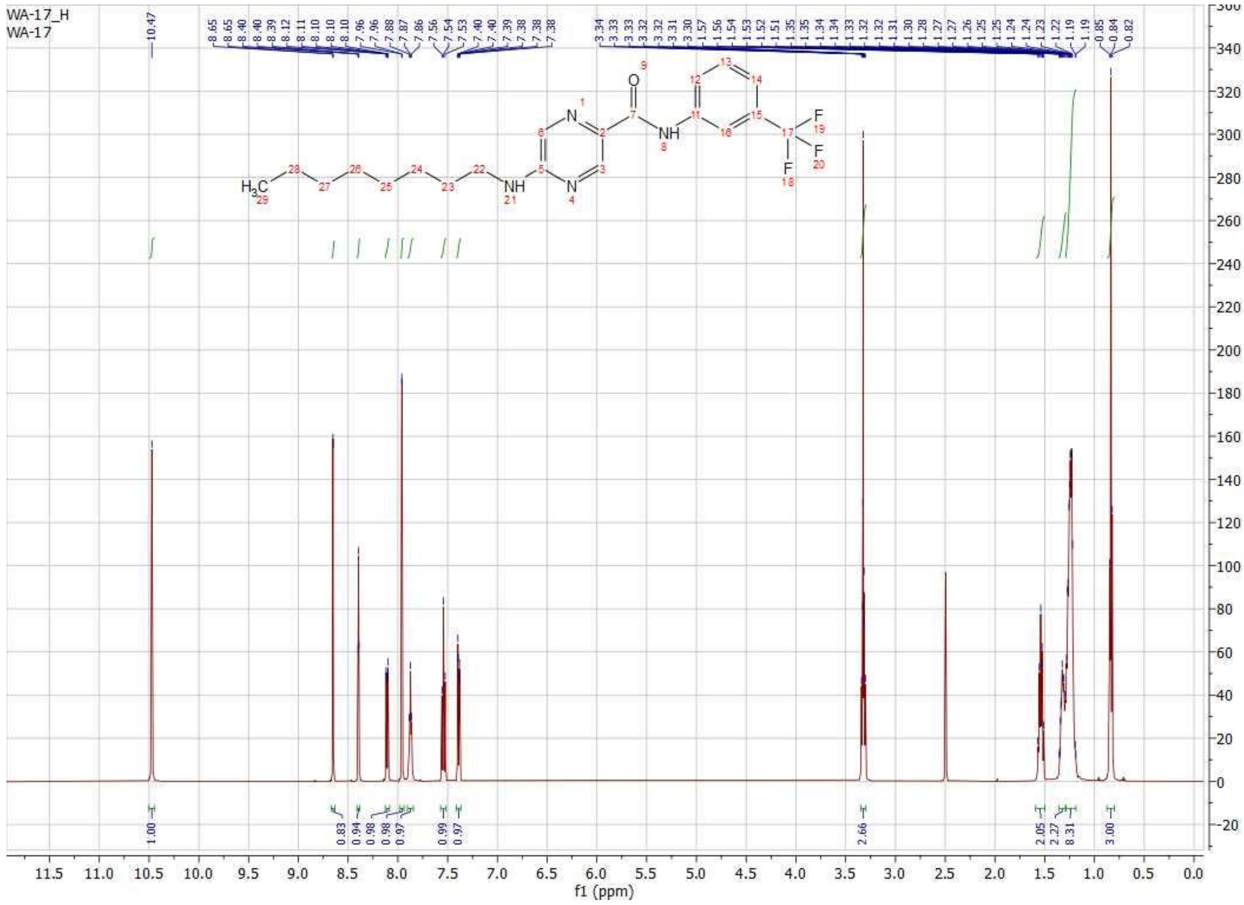

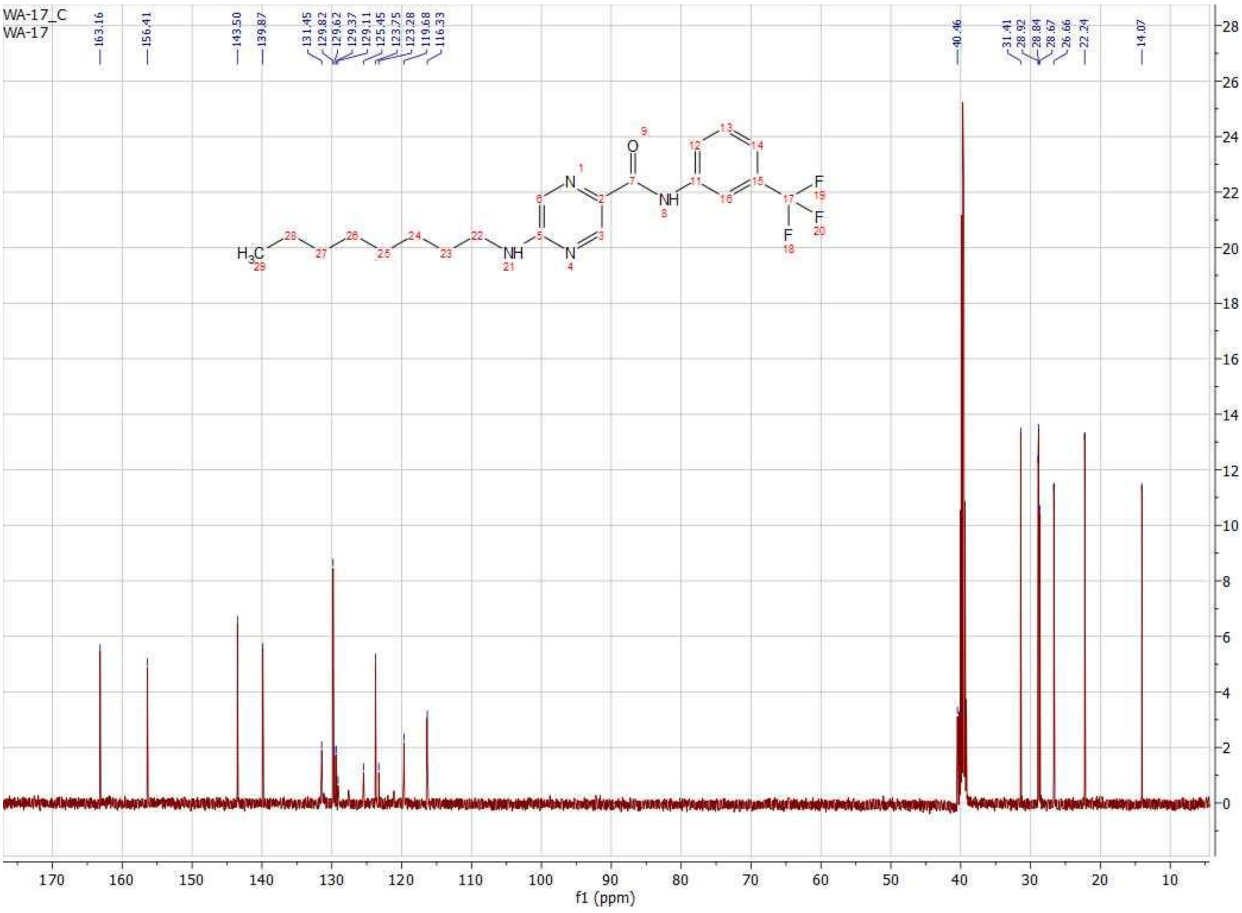

^{1}H and ^{13}C NMR spectra of compound 2a

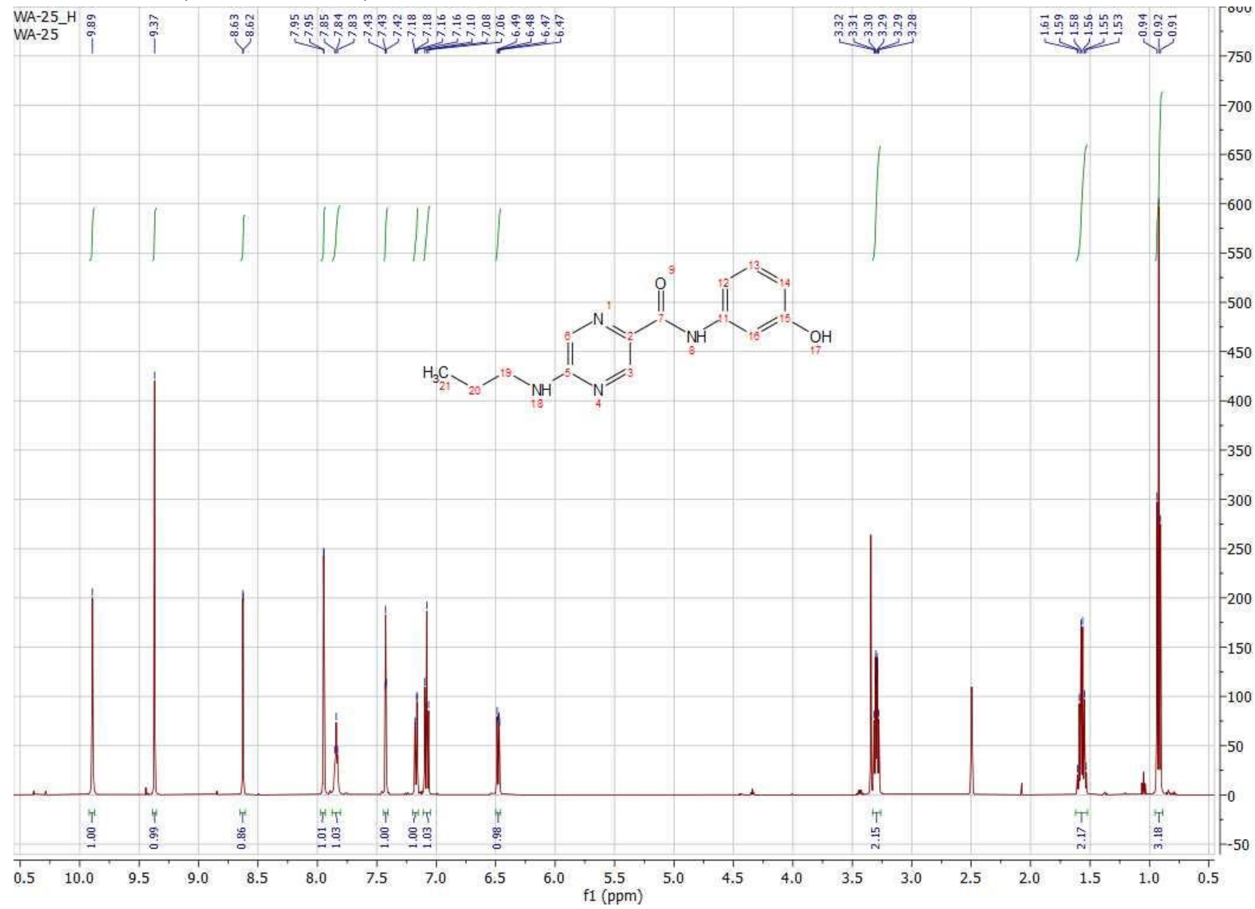

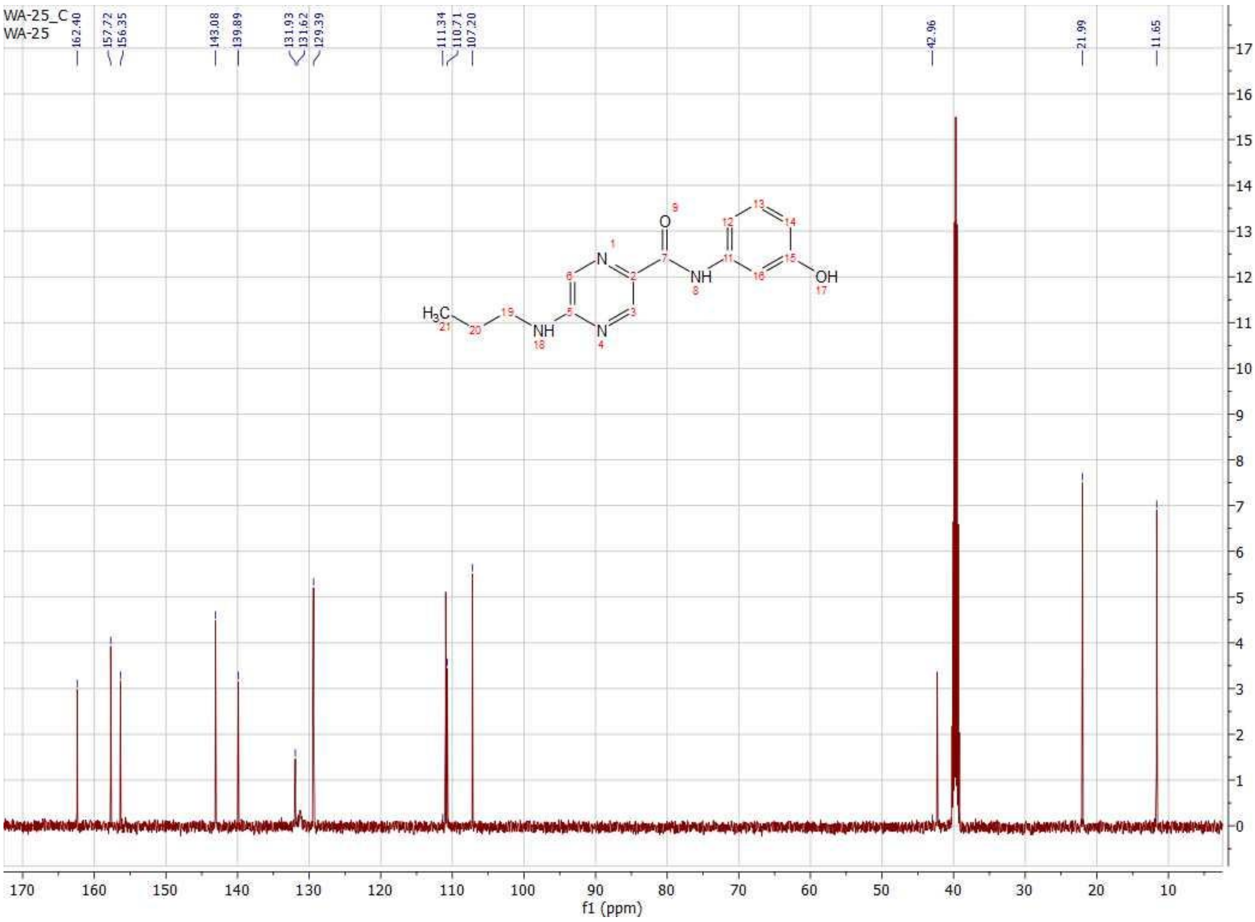

^{1}H and ^{13}C NMR spectra of compound 2d

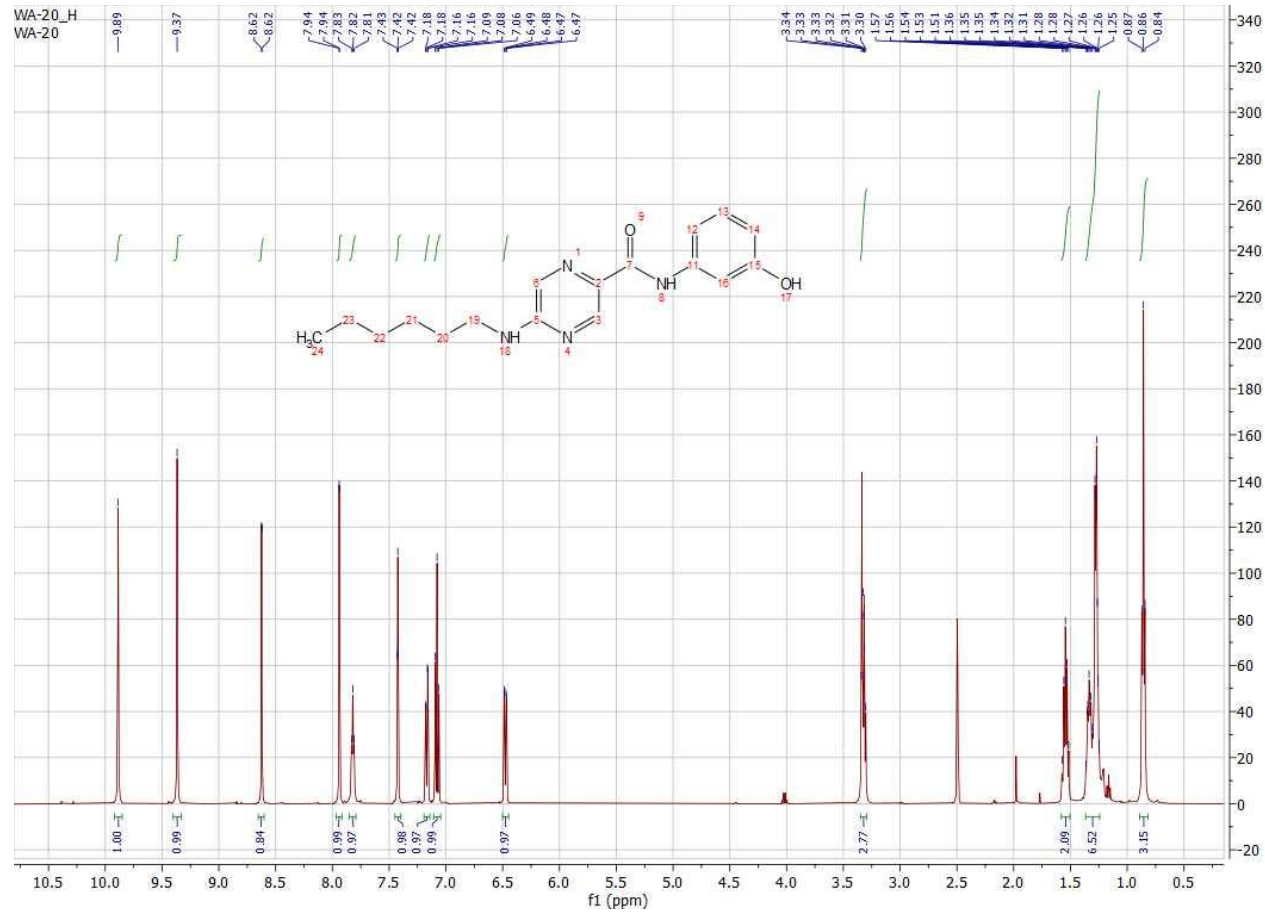

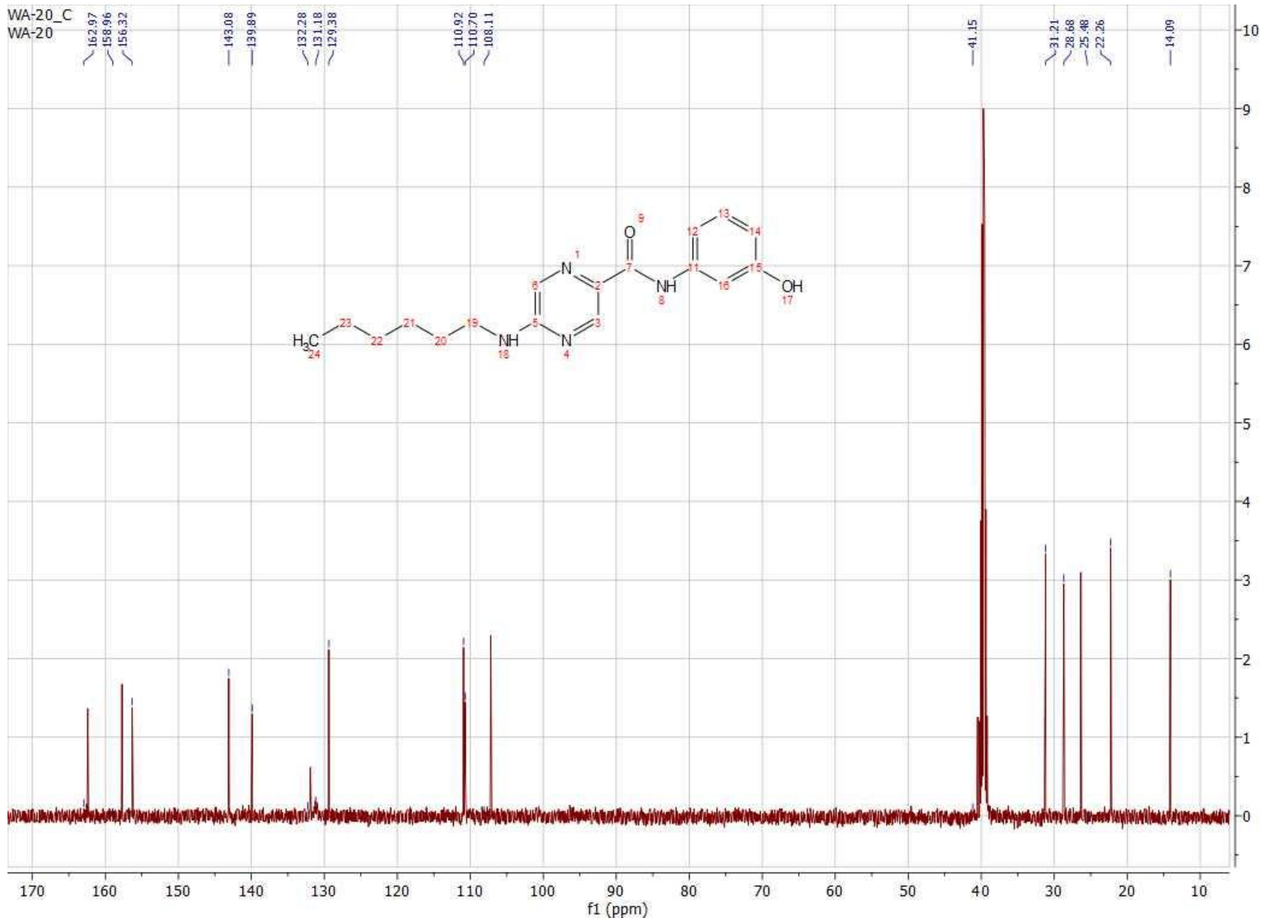

^{1}H and ^{13}C NMR spectra of compound 3b

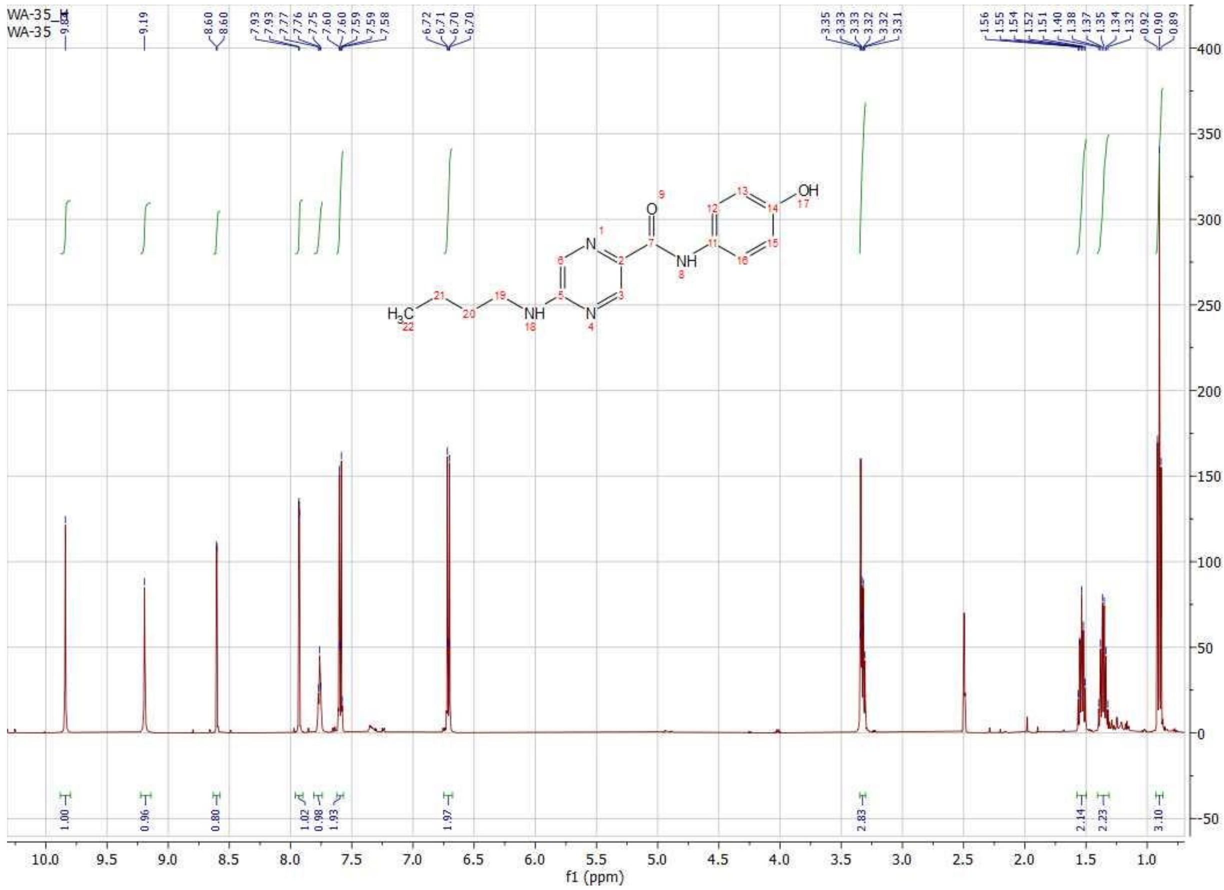

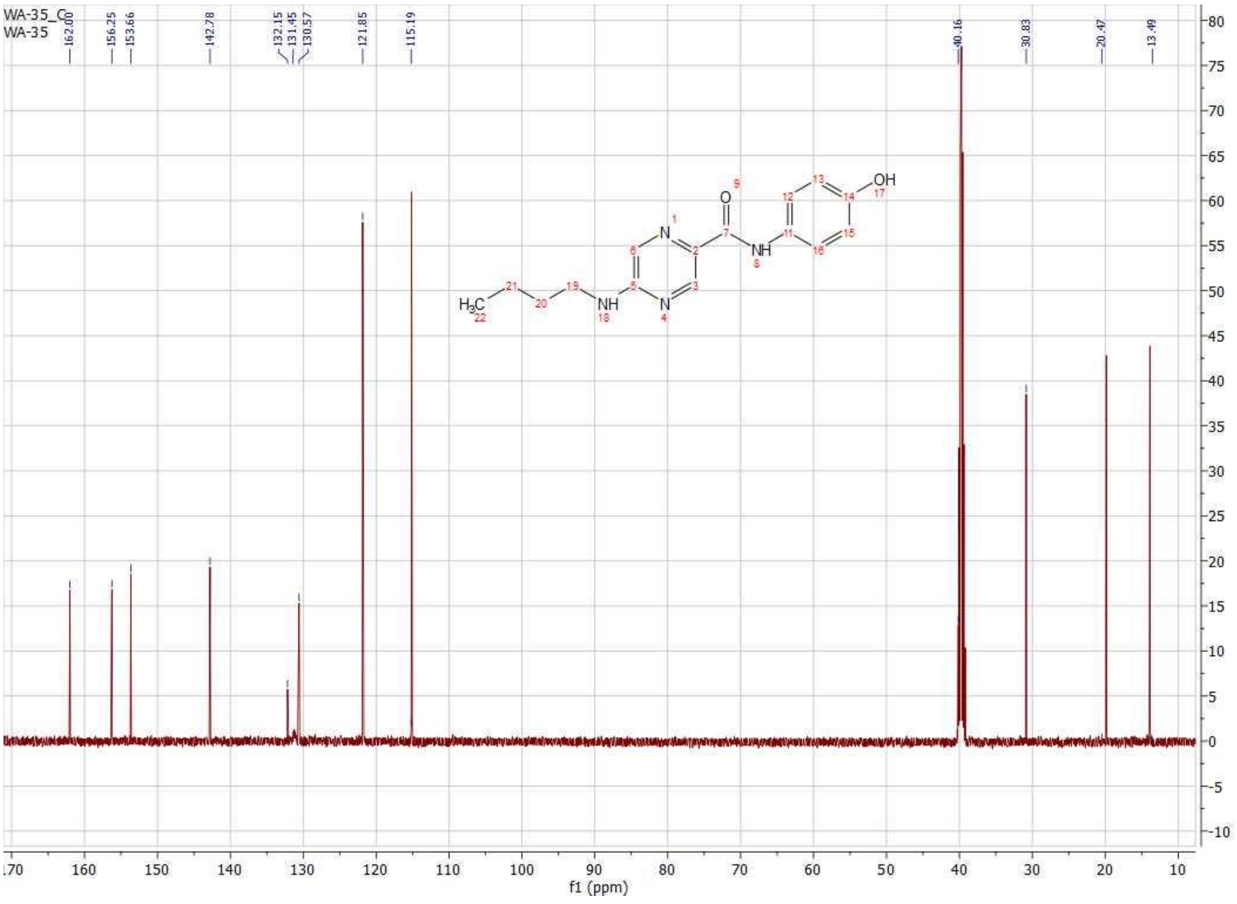

^{1}H and ^{13}C NMR spectra of compound 3f

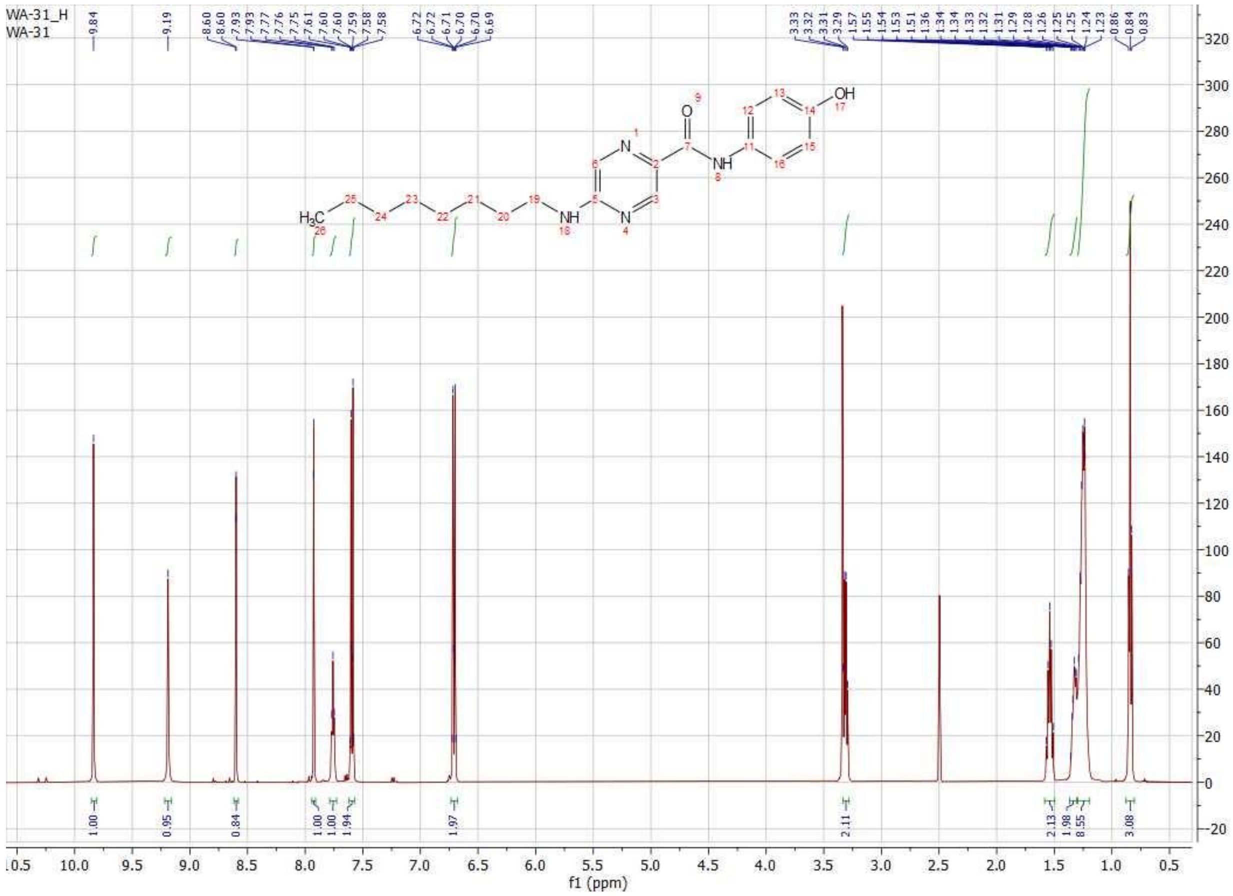

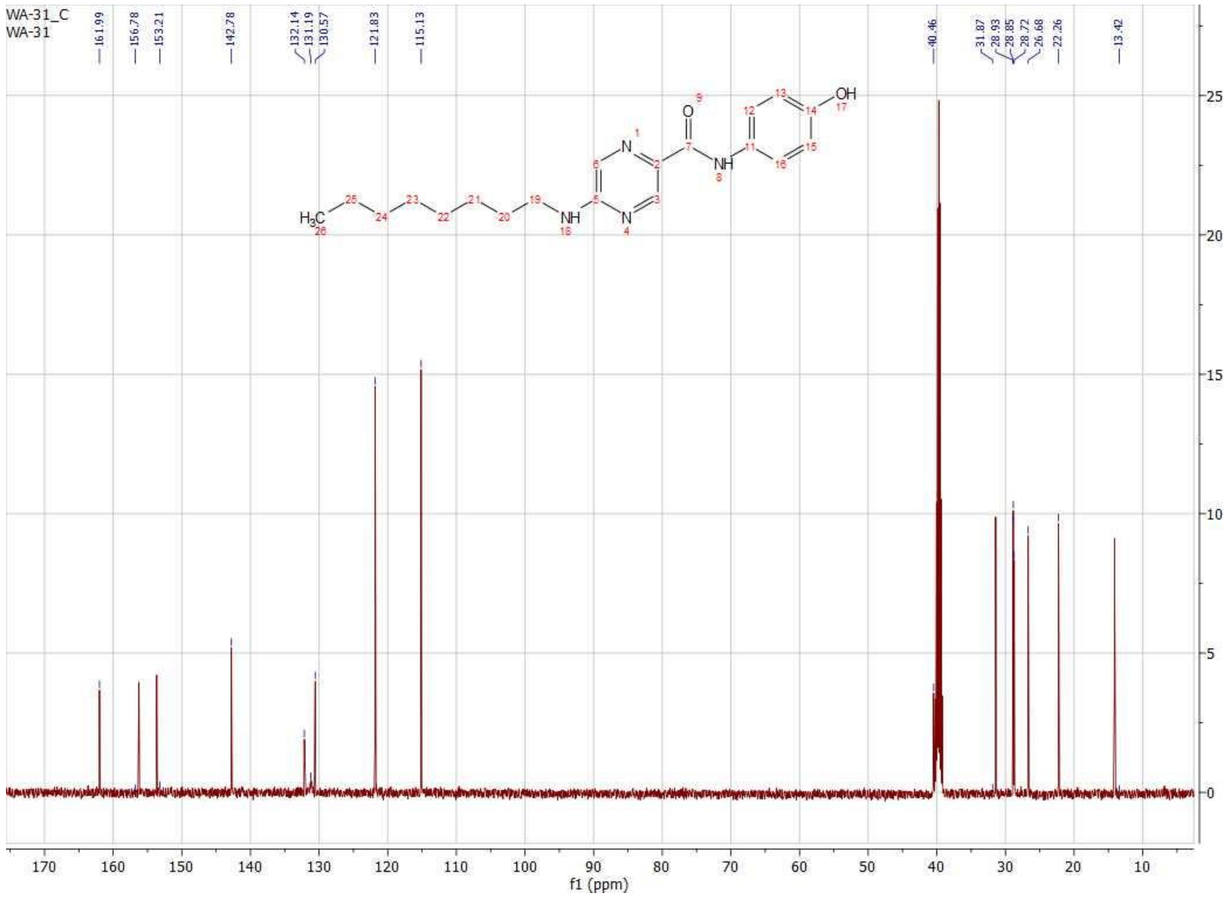

^{1}H and ^{13}C NMR spectra of compound 4a

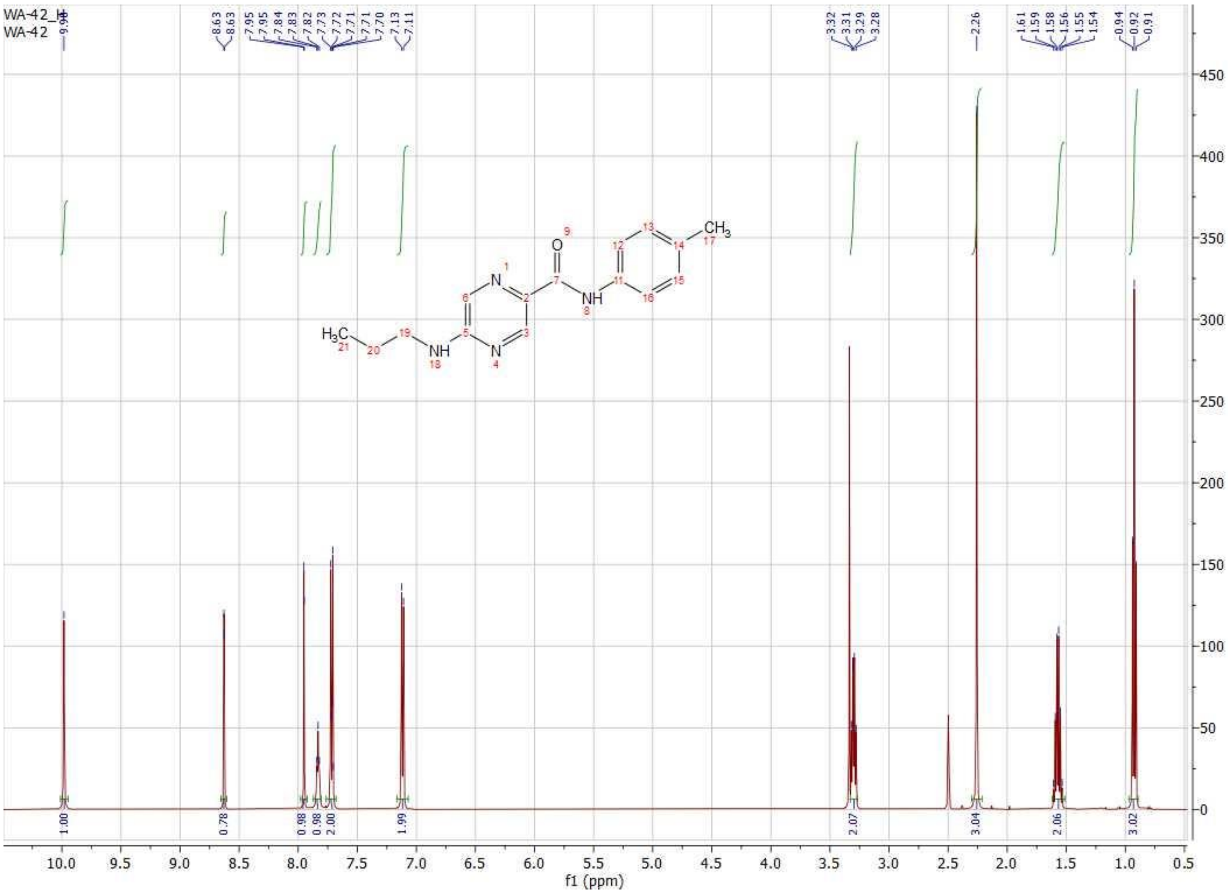

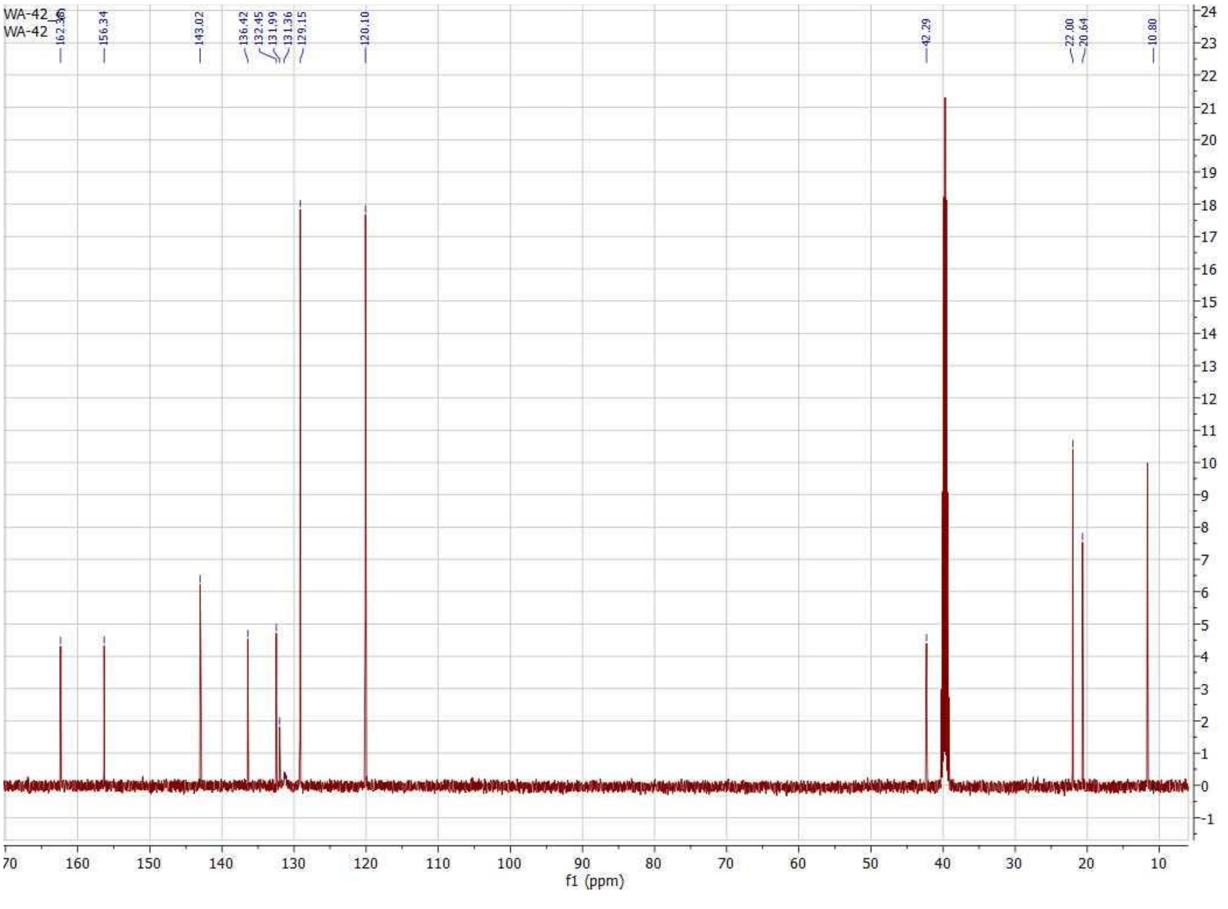

^{1}H and ^{13}C NMR spectra of compound 4d

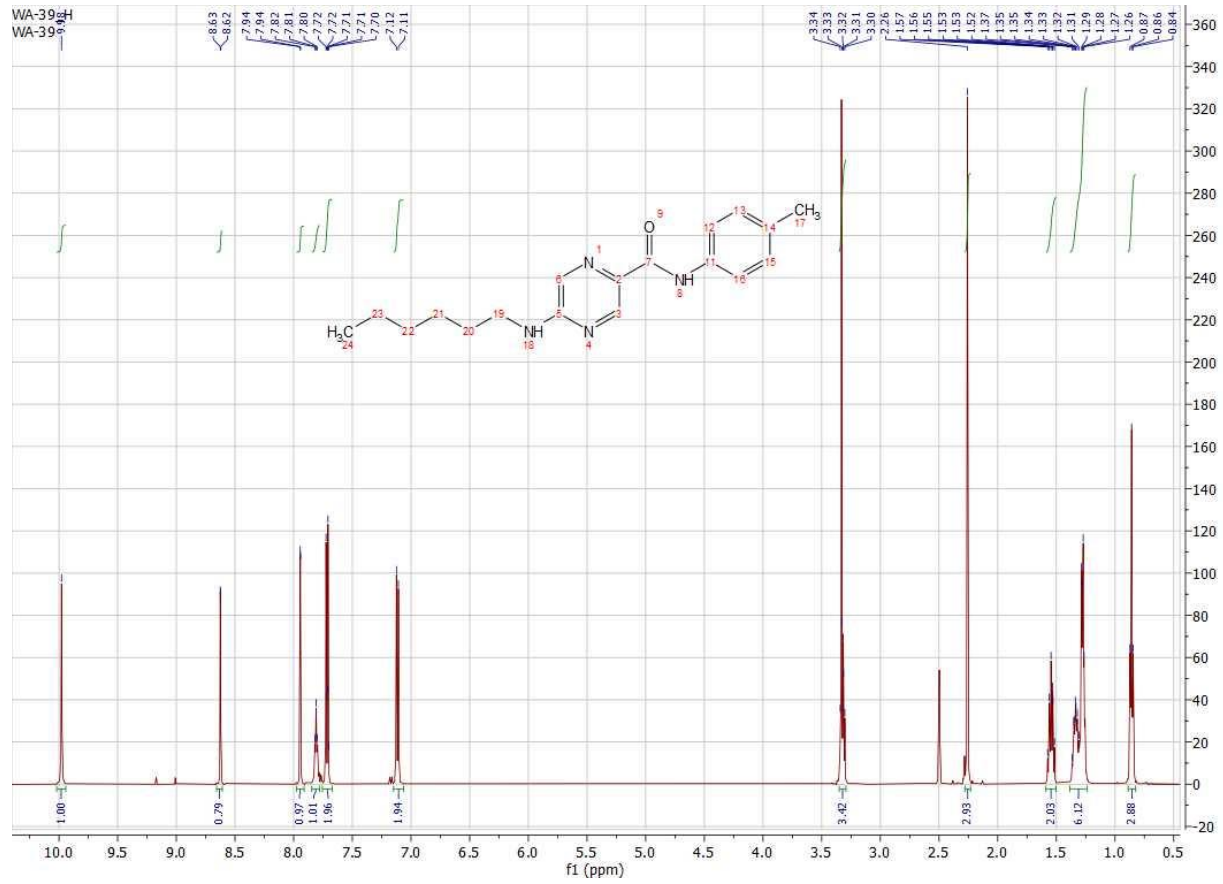

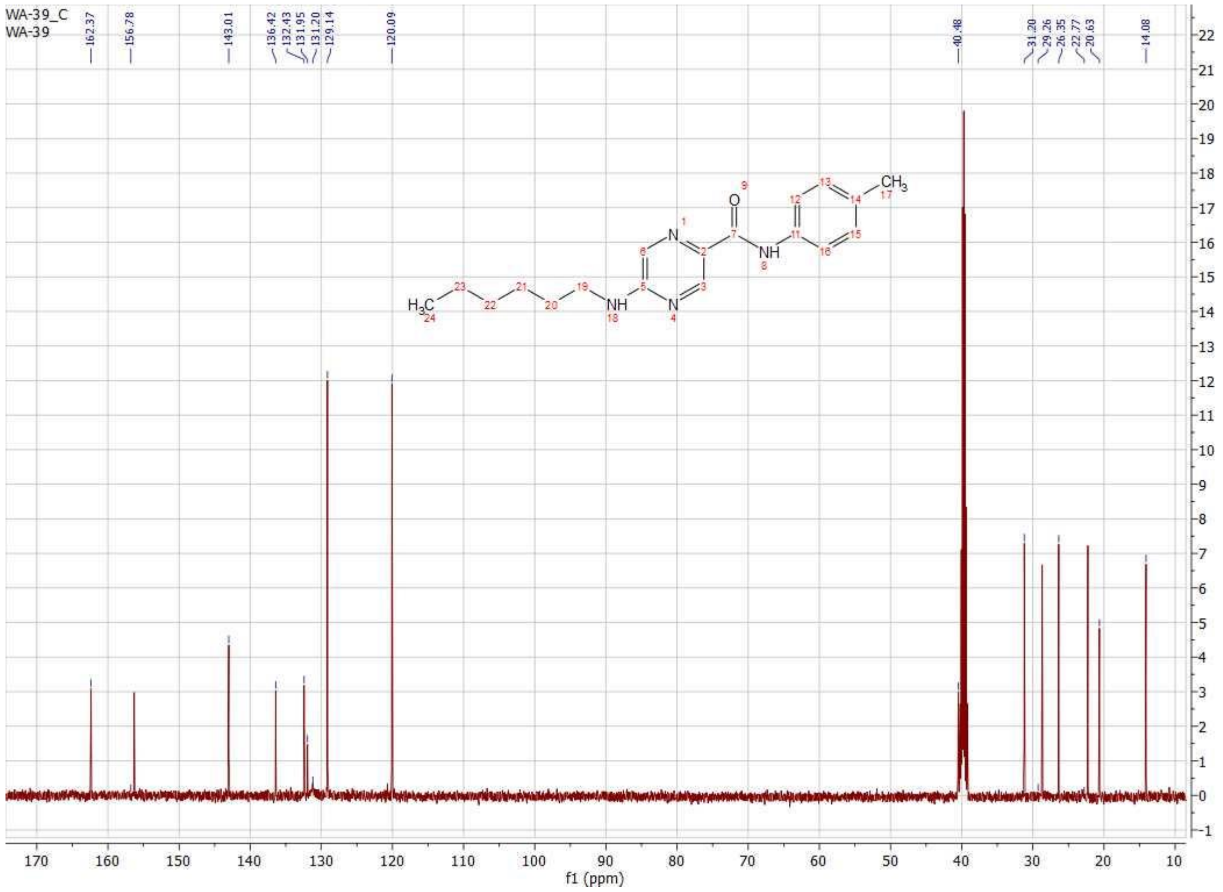

^{1}H and ^{13}C NMR spectra of compound 5a

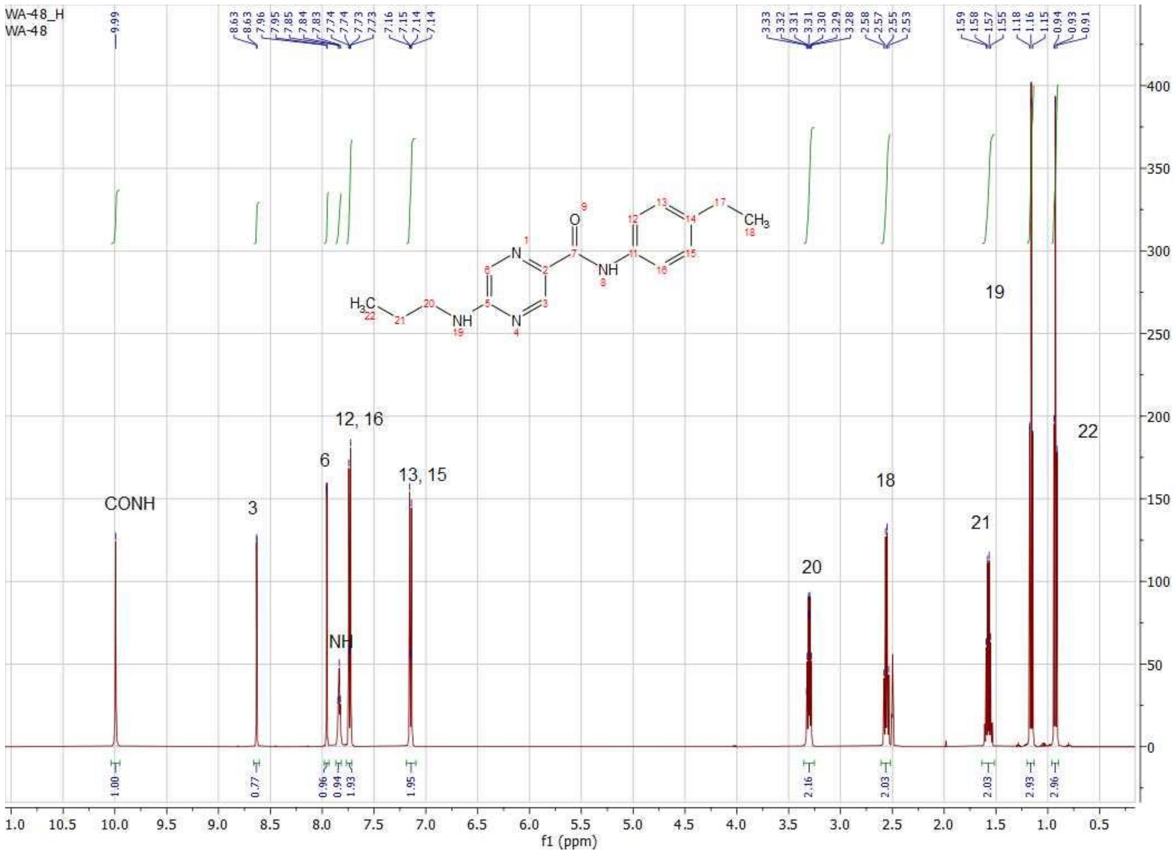

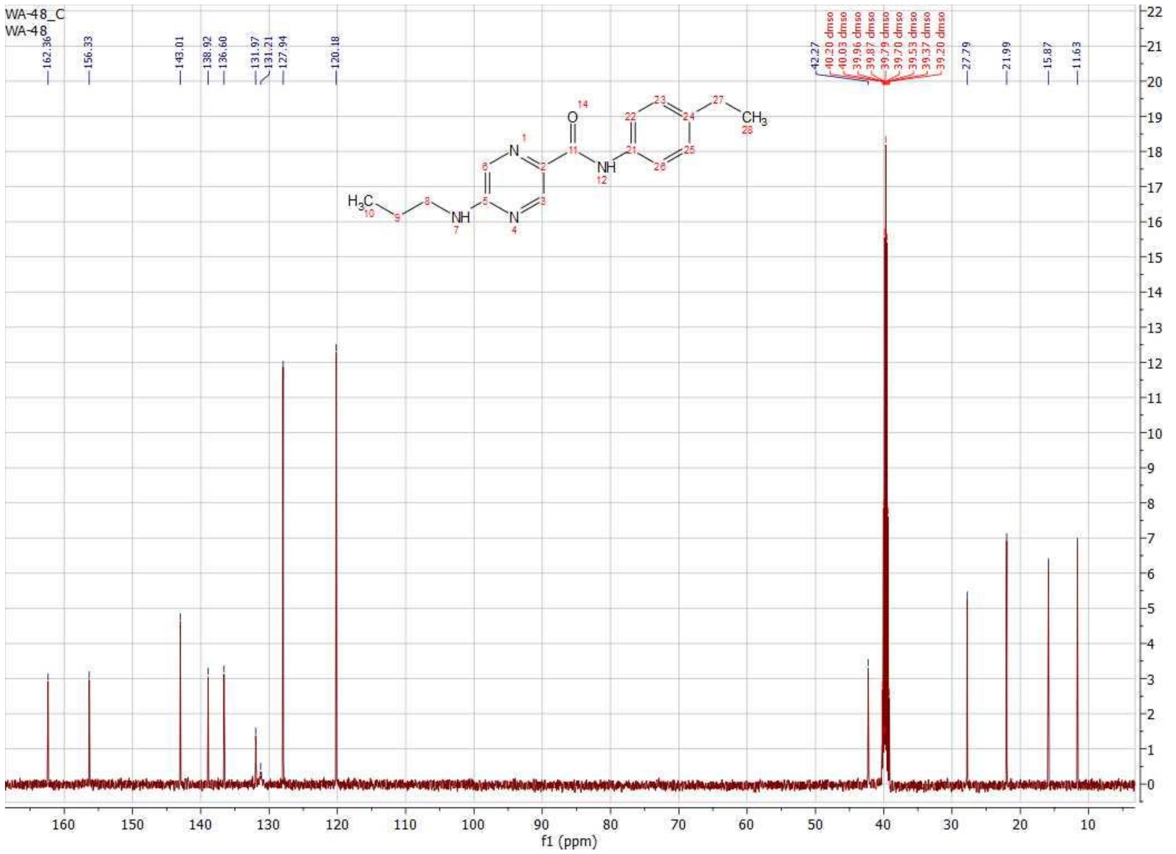

^{1}H and ^{13}C NMR spectra of compound 5e

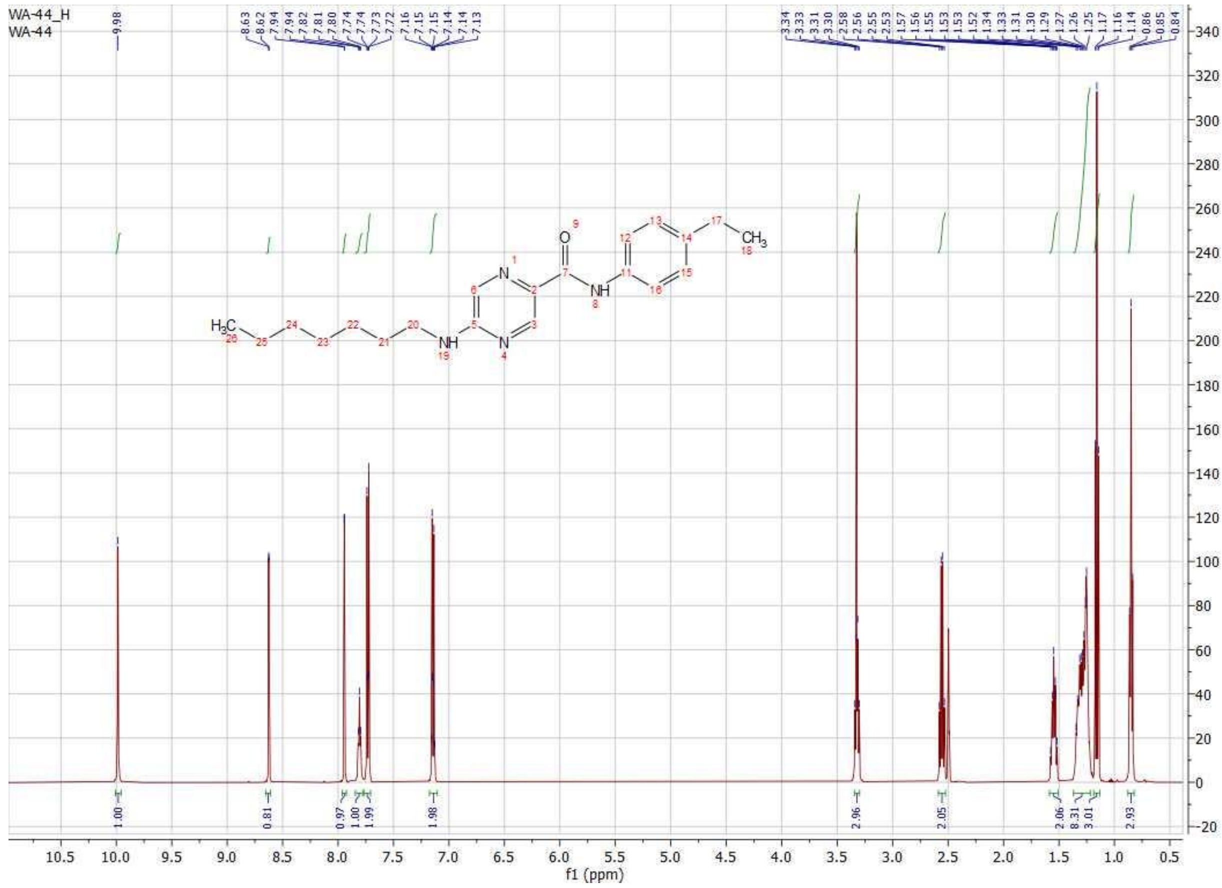

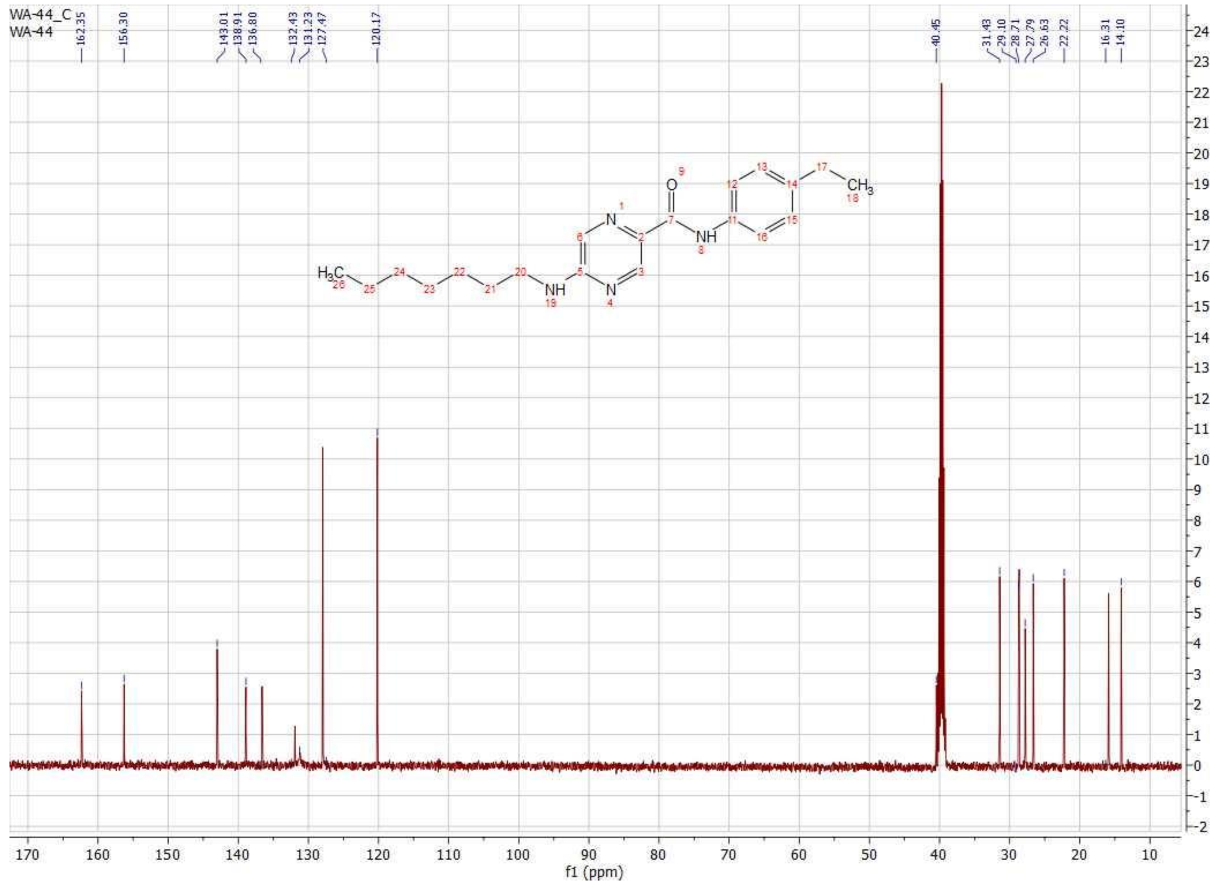

Full results of antibacterial activity

Not all compounds were evaluated due to a problem with solubility which occurred for eighteen samples. Compounds **2c**, **2d**, **2e**, **4a**, **5b**, **5c** and **5f** precipitated in cultivation media and compounds **3f**, **6c**, **6d**, **6e**, **6f**, **7a**, **7b**, **7c**, **7d**, **7e** and **7f** did not dissolve in DMSO when preparing the stock solution.

Tested strains (code, number)	
1. **SA** *Staphylococcus aureus* spp.*aureus*	ATCC 29213, CCM 4223
2. **MRSA** *Staphylococcus aureus* spp.*aureus*	ATCC 43300, CCM 4750
3. **SE** *Staphylococcus epidermidis*	ATCC 12228, CCM 4418
4. **EF** *Enterococcus faecalis*	ATCC 29212, CCM 4224
5. **EC** *Escherichia coli*	ATCC 25922, CCM 3954
6. **KP** *Klebsiella pneumoniae*	ATCC 10031, CCM 4415
7. **ACI** *Acinetobacter baumanii*	ATCC 19606, DSM 30007
8. **PA** *Pseudomonas aeruginosa*	ATCC 27853, CCM 3955

Results

**STRAIN** (code)		**TESTED COMPOUND** (lab code, code in publication) – **MIC** (μM)									
		**WA-19 1a**	**WA-18 1b**	**WA-15 1c**	**WA_14 1d**	**WA-16 1e**	**WA-17 1f**	**WA-24 2b**	**WA-25 2a**	**WA-23 2f**	
**SA**	24h	>500	>500	>500	>500	>500	>500	>500	>125	>125	
	48h	>500	>500	>500	>500	>500	>500	>500	>125	>125	
**MRSA**	24h	>500	>500	>500	>500	>500	>500	>500	>125	>125	
	48h	>500	>500	>500	>500	>500	>500	>500	>125	>125	
**SE**	24h	>500	>500	>500	>500	>500	>500	>500	>125	>125	
	48h	>500	>500	>500	>500	>500	>500	>500	>125	>125	
**EF**	24h	>500	>500	>500	>500	>500	>500	>500	>125	>125	
	48h	>500	>500	>500	>500	>500	>500	>500	>125	>125	
**EC**	24h	>500	>500	>500	>500	>500	>500	>500	>125	>125	
	48h	>500	>500	>500	>500	>500	>500	>500	>125	>125	
**KP**	24h	>500	>500	>500	>500	>500	>500	>500	>125	>125	
	48h	>500	>500	>500	>500	>500	>500	>500	>125	>125	
**ACI**	24h	>500	>500	>500	>500	>500	>500	>500	>125	>125	
	48h	>500	>500	>500	>500	>500	>500	>500	>125	>125	
**PA**	72h	>500	>500	>500	>500	>500	>500	>500	>125	>125	
	120h	>500	>500	>500	>500	>500	>500	>500	>125	>125	

STRAIN (code)		TESTED COMPOUND (lab code, code in publication) – MIC (μM)									
		**WA-36 3a**	**WA-35 3b**	**WA-34 3c**	**WA-33 3d**	**WA-32 3e**	**WA-41 4b**	**WA-40 4c**	**WA-39 4d**	**WA-38 4e**	**WA-37 4f**
**SA**	24h	>500	>500	>125	>500	>500	>125	>500	>500	>500	>125
	48h	>500	>500	>125	>500	>500	>125	>500	>500	>500	>125
**MRSA**	24h	>500	>500	>125	>500	>500	>125	>500	>500	>500	>125
	48h	>500	>500	>125	>500	>500	>125	>500	>500	>500	>125
**SE**	24h	>500	>500	>125	>500	>500	>125	>500	>500	>500	>125
	48h	>500	>500	>125	>500	>500	>125	>500	>500	>500	>125
**EF**	24h	>500	>500	>125	>500	>500	>125	>500	>500	>500	>125
	48h	>500	>500	>125	>500	>500	>125	>500	>500	>500	>125
**EC**	24h	>500	>500	>125	>500	>500	>125	>500	>500	>500	>125
	48h	>500	>500	>125	>500	>500	>125	>500	>500	>500	>125
**KP**	24h	>500	>500	>125	>500	>500	>125	>500	>500	>500	>125
	48h	>500	>500	>125	>500	>500	>125	>500	>500	>500	>125
**ACI**	24h	>500	>500	>125	>500	>500	>125	>500	>500	>500	>125
	48h	>500	>500	>125	>500	>500	>125	>500	>500	>500	>125
**PA**	72h	>500	>500	>125	>500	>500	>125	>500	>500	>500	>125
	120h	>500	>500	>125	>500	>500	>125	>500	>500	>500	>125

Results

**STRAIN** (code)		**TESTED COMPOUND** (lab code, code in publication) – **MIC** (μM)									
		**WA-48 5a**	**WA-45 5d**	**WA-44 5e**							
**SA**	24h	>125	>500	>500							
	48h	>125	>500	>500							
**MRSA**	24h	>125	>500	>500							
	48h	>125	>500	>500							
**SE**	24h	>125	>500	>500							
	48h	>125	>500	>500							
**EF**	24h	>125	>500	>500							
	48h	>125	>500	>500							
**EC**	24h	>125	>500	>500							
	48h	>125	>500	>500							
**KP**	24h	>125	>500	>500							
	48h	>125	>500	>500							
**ACI**	24h	>125	>500	>500							
	48h	>125	>500	>500							
**PA**	72h	>125	>500	>500							
	120h	>125	>500	>500							

Full results of antifungal activity

Not all compounds were evaluated due to a problem with solubility which occurred for eighteen samples. Compounds **2c**, **2d**, **2e**, **4a**, **5b**, **5c** and **5f** precipitated in cultivation media and compounds **3f**, **6c**, **6d**, **6e**, **6f**, **7a**, **7b**, **7c**, **7d**, **7e** and **7f** did not dissolve in DMSO when preparing the stock solution.

Tested strains (code, number)	
1. **CA** -*Candida albicans*	ATCC 24433, CCM 8320
2. **CK** - *Candida krusei*	ATCC 6258, CCM 8271
3. **CP** - *Candida parapsilosis*	ATCC 22019, CCM 8260
4. **CT** -*Candida tropicalis*	ATCC 750, CCM 8264
5. **AF** -*Aspergillus fumigatus*	ATCC 204305
6. **AFla-** *Aspergillus flavus*	CCM 8363
7. **AC** -*Absidia corymbifera*	CCM 8077
8. **TI** -*Trichophyton interdigitale*	ATCC 9533, CCM 8377

Results

**STRAIN** (code)		**TESTED COMPOUND** (lab code, code in publication) – **MIC** (µM)								
		**WA-19 1a**	**WA-18 1b**	**WA-15 1c**	**WA_14 1d**	**WA-16 1e**	**WA-17 1f**	**WA-25 2a**	**WA-24 2b**	**WA-23 2f**
**CA**	24h	>125	>125	>125	>500	>500	>500	>125	>125	>125
	48h	>125	>125	>125	>500	>500	>500	>125	>125	>125
**CK**	24h	>125	>125	**62.5**	>500	>500	>500	**62.5**	>125	>125
	48h	>125	>125	**62.5**	>500	>500	>500	**62.5**	>125	>125
**CP**	24h	>125	>125	**125**	>500	>500	>500	>125	>125	>125
	48h	>125	>125	**125**	>500	>500	>500	>125	>125	>125
**CT**	24h	>125	>125	>125	>500	>500	>500	>125	>125	>125
	48h	>125	>125	>125	>500	>500	>500	>125	>125	>125
**AF**	24h	>125	>125	>125	>500	>500	>500	>125	>125	>125
	48h	>125	>125	>125	>500	>500	>500	>125	>125	>125
**AFla**	24h	>125	>125	>125	>500	>500	>500	>125	>125	>125
	48h	>125	>125	>125	>500	>500	>500	>125	>125	>125
**AC**	24h	>125	>125	>125	>500	>500	>500	>125	>125	>125
	48h	>125	>125	>125	>500	>500	>500	>125	>125	>125
**TI**	72h	>125	>125	>125	>500	>500	>500	>125	>125	>125
	120h	>125	>125	>125	>500	>500	>500	>125	>125	>125

STRAIN (code)		TESTED COMPOUND (lab code, code in publication) – MIC (μM)									
		**WA-36 3a**	**WA-35 3b**	**WA-34 3c**	**WA-33 3d**	**WA-32 3e**	**WA-41 4b**	**WA-40 4c**	**WA-39 4d**	**WA-38 4e**	**WA-37 4f**
**CA**	24h	>500	**62,5**	**62,5**	>500	>250	>125	>125	>125	>125	>125
	48h	>500	**62,5**	**62,5**	>500	>250	>125	>125	>125	>125	>125
**CK**	24h	>500	>250	>125	>500	>250	>125	>125	**125**	>125	>125
	48h	>500	>250	>125	>500	>250	>125	>125	**125**	>125	>125
**CP**	24h	>500	**250**	>125	>500	>250	>125	>125	**125**	>125	>125
	48h	>500	**250**	>125	>500	>250	>125	>125	**125**	>125	>125
**CT**	24h	>500	>250	>125	>500	>250	>125	>125	>125	>125	>125
	48h	>500	>250	>125	>500	>250	>125	>125	>125	>125	>125
**AF**	24h	>500	>250	>125	>500	>250	>125	>125	>125	>125	>125
	48h	>500	>250	>125	>500	>250	>125	>125	>125	>125	>125
**AFla**	24h	>500	>250	>125	>500	>250	>125	>125	>125	>125	>125
	48h	>500	>250	>125	>500	>250	>125	>125	>125	>125	>125
**AC**	24h	>500	>250	>125	>500	>250	>125	>125	>125	>125	>125
	48h	>500	>250	>125	>500	>250	>125	>125	>125	>125	>125
**TI**	72h	>500	>250	>125	>500	>250	>125	>125	>125	>125	>125
	120h	>500	>250	>125	>500	>250	>125	>125	>125	>125	>125

**STRAIN** (code)		**TESTED COMPOUND** (lab code, code in publication) – **MIC** (μM)									
		**WA-48 5a**	**WA-45 5d**	**WA-44 5e**							
**CA**	24h	>125	>125	>125							
	48h	>125	>125	>125							
**CK**	24h	>125	>125	>125							
	48h	>125	>125	>125							
**CP**	24h	>125	>125	>125							
	48h	>125	>125	>125							
**CT**	24h	>125	>125	>125							
	48h	>125	>125	>125							
**AF**	24h	>125	>125	>125							
	48h	>125	>125	>125							
**AFla**	24h	>125	>125	>125							
	48h	>125	>125	>125							
**AC**	24h	>125	>125	>125							
	48h	>125	>125	>125							
**TI**	72h	>125	>125	>125							
	120h	>125	>125	>125							

References

1. European Committee for Antimicrobial Susceptibility Testing (EUCAST) of the European Society of Clinical Microbiology and Infectious Diseases (ESCMID). EUCAST DISCUSSION DOCUMENT E.Dis 5.1. Determination of minimum inhibitory concentrations (MICs) of antibacterial agents by broth dilution. *Clin. Microbiol. Infect.* **2003**, *9*, 1–7. doi: 10.1046/j.1469-0691.2003.00790.x.
2. Arendrup, M.C.; Meletiadis, J.; Mouton, J.W.; Lagrou, K.; Hamal, P.; Guinea, J.; Subcommittee on Antifungal Susceptibility Testing (AFST) of the ESCMID European Committee for Antimicrobial Susceptibility Testing (EUCAST). Method for the determination of broth dilution minimum inhibitory concentrations of antifungal agents for yeasts. EUCAST Definitive Document E.Def 7.3.1. 2017. Available online: http://www.eucast.org/astoffungi/methodsinantifungalsusceptibilitytesting/susceptibility_testing_of_yeasts/ (accessed on 5 March 2020).
3. Arendrup, M.C.; Meletiadis, J.; Mouton, J.W.; Lagrou, K.; Hamal, P.; Guinea, J.; Subcommittee on Antifungal Susceptibility Testing (AFST) of the ESCMID European Committee for Antimicrobial Susceptibility Testing (EUCAST). Method for the determination of broth dilution minimum inhibitory concentrations of antifungal agents for conidia forming moulds. EUCAST Definitive Document E.Def 9.3.1. 2017. Available online: http://www.eucast.org/astoffungi/methodsinantifungalsusceptibilitytesting/susceptibility_testing_of_moulds/ (accessed on 5 March 2020).
